# FOXG1 Regulates PRKAR2B Transcriptionally and Posttranscriptionally via miR200 in the Adult Hippocampus

**DOI:** 10.1007/s12035-018-1444-7

**Published:** 2018-12-11

**Authors:** Stefan C. Weise, Ganeshkumar Arumugam, Alejandro Villarreal, Pavankumar Videm, Stefanie Heidrich, Nils Nebel, Verónica I. Dumit, Farahnaz Sananbenesi, Viktoria Reimann, Madeline Craske, Oliver Schilling, Wolfgang R. Hess, Andre Fischer, Rolf Backofen, Tanja Vogel

**Affiliations:** 1 Institute of Anatomy and Cell Biology, Department of Molecular Embryology, Faculty of Medicine, Albert-Ludwigs-University Freiburg, 79104 Freiburg, Germany; 2 Faculty of Biology, Albert-Ludwigs-University Freiburg, 79104 Freiburg, Germany; 3 Institute of Anatomy and Cell Biology, Department of Neuroanatomy, Faculty of Medicine, Albert-Ludwigs-University Freiburg, 79104 Freiburg, Germany; 4 Spemann Graduate School of Biology and Medicine, Albert-Ludwigs-University Freiburg, 79104 Freiburg, Germany; 5 Bioinformatics Group, Department of Computer Science, Albert-Ludwigs-University Freiburg, 79110 Freiburg, Germany; 6 Centre for Biological Systems Analysis (ZBSA), Albert-Ludwigs-University Freiburg, 79104 Freiburg, Germany; 7 Group for Genome Dynamics in Neurodegenerative Diseases, German Center for Neurodegenerative Diseases (DZNE), 37075 Göttingen, Germany; 8 Genetics and Experimental Bioinformatics, Faculty of Biology, Albert-Ludwigs-University Freiburg, 79104 Freiburg, Germany; 9 Active Motif Incorporation, Carlsbad, CA USA; 10 Centre for Biological Signalling Studies (BIOSS), Albert-Ludwigs-University Freiburg, 79104 Freiburg, Germany; 11 Institute of Molecular Medicine and Cell Research, Faculty of Medicine, Albert-Ludwigs-University Freiburg, 79104 Freiburg, Germany; 12 Freiburg Institute for Advanced Studies, Albert-Ludwigs-University Freiburg, 79104 Freiburg, Germany; 13 Department for Epigenetics and Systems Medicine in Neurodegenerative Diseases, German Center for Neurodegenerative Diseases (DZNE), 37075 Göttingen, Germany; 14 Department for Psychiatry and Psychotherapy, University Medical Center, Georg August University Göttingen, 37075 Göttingen, Germany; 15 Center for non-coding RNA in Technology and Health, University of Copenhagen, 1870 Frederiksberg C, Denmark

**Keywords:** DROSHA, Atypical Rett syndrome, MECP2, Neurogenesis, PKA

## Abstract

Rett syndrome is a complex neurodevelopmental disorder that is mainly caused by mutations in *MECP2*. However, mutations in *FOXG1* cause a less frequent form of atypical Rett syndrome, called FOXG1 syndrome. FOXG1 is a key transcription factor crucial for forebrain development, where it maintains the balance between progenitor proliferation and neuronal differentiation. Using genome-wide small RNA sequencing and quantitative proteomics, we identified that FOXG1 affects the biogenesis of miR200b/a/429 and interacts with the ATP-dependent RNA helicase, DDX5/p68. Both FOXG1 and DDX5 associate with the microprocessor complex, whereby DDX5 recruits FOXG1 to DROSHA. RNA-Seq analyses of *Foxg1*^cre/+^ hippocampi and N2a cells overexpressing miR200 family members identified cAMP-dependent protein kinase type II-beta regulatory subunit (PRKAR2B) as a target of miR200 in neural cells. PRKAR2B inhibits postsynaptic functions by attenuating protein kinase A (PKA) activity; thus, increased PRKAR2B levels may contribute to neuronal dysfunctions in FOXG1 syndrome. Our data suggest that FOXG1 regulates PRKAR2B expression both on transcriptional and posttranscriptional levels.

**Electronic supplementary material:**

The online version of this article (10.1007/s12035-018-1444-7) contains supplementary material, which is available to authorized users.

## Introduction

Rett syndrome (RTT) is a progressive neurodevelopmental disorder that affects one in 10,000 females, and it is the second leading cause of female intellectual deficiency. RTT patients show symptoms such as microcephaly, seizures, indifference to visual/auditory stimuli, and severe cognitive dysfunction [1]. There are two forms of RTT, namely typical RTT (tRTT) and atypical RTT-like (atRTT). About 70–90% of cases are due to tRTT, which results from mutations in the Methyl CpG binding Protein 2 (*MECP2*) gene. Different subvariants of atRTT have been defined, one of which is caused by mutations in the Forkhead box G1 (*FOXG1*) gene (FOXG1 syndrome, OMIM#164874). Surprisingly, both loss- and gain-of-function mutations result in clinical phenotypes, which encompass common (e.g. seizures) but also unique features (e.g. spasms). Rett-like syndrome and epilepsy have been associated with *FOXG1*-haploinsufficiency [2]. Loss- and gain-of-function mutations are reported also for both tRTT and atRTT, whereby gain-of-function is caused by gene duplication of either *MECP2* or *FOXG1* [3, 4]. Therefore, both gene products seem to be associated with functions that are dosage sensitive, although these functions are so far ill defined, especially for FOXG1.

FOXG1 plays a central role in forebrain development as its complete absence results in anencephaly [5]. FOXG1 influences proliferation as well as differentiation of neural stem cells, and it is involved in migration and integration of pyramidal neurons into the cortical plate [6]. FOXG1-deficient stem cells differentiate prematurely to Cajal–Retzius neurons, whereas overexpression of FOXG1 increases the stem cell pool and delays neurogenesis [7, 8]. On a molecular level, FOXG1 represses expression of cyclin-dependent kinase inhibitor 1A (*Cdkn1a*) and thereby prevents cell cycle exit of progenitor cells and promotes stem cell pool expansion [9, 10]. Cell cycle regulation through FOXG1 is mediated by its binding to Forkhead box O− (FOXO−) and SMAD− (SMA and MAD related−) protein complexes and by antagonising TGFβ-induced neuronal differentiation [11]. In addition, FOXG1 deficiency results in the loss of the ventral telencephalon through impaired expression of ventralising signals [12]. Also, FOXG1 interacts with one of two MECP2-isoforms (MECP2-e2), which prevents cell death of cerebellar neurons [13]. Several mouse models were used to study the molecular basis of tRTT and atRTT, and some of these studies included non-coding RNA (ncRNA), such as miRNAs. Whereas altered expression of ncRNA is involved in *MECP2*-mediated RTT [14–16], a comprehensive expression study of the misregulated coding and non-coding transcriptome is missing for FOXG1 haploinsufficient adult brains.

Here, we report on altered expression of members of the miRNA200 family, namely miR200a, miR200b and miR429 in the adult *Foxg1*^*cre/+*^ hippocampus. Stable isotope labelling with amino acids in cell culture (SILAC) followed by quantitative mass spectrometry revealed that FOXG1 associates with the RNA helicase DDX5 (DEAD (Asp-Glu-Ala-Asp) box polypeptide 5, p68). DDX5 recruits FOXG1 to the DROSHA complex, and FOXG1 overexpression alongside with reduced levels of DDX5 affects biogenesis of miR200 family members. Decreased expression of FOXG1 and overexpression of miR200 result in altered expression levels of protein kinase cAMP-dependent regulatory type II beta (PRKAR2B)*.* As PRKAR2B influences synaptic function, our results reveal a novel candidate gene, whose altered expression might be implicated in FOXG1 syndrome. Additionally, we establish that FOXG1 has functions in posttranscriptional regulation besides its known role as transcription factor [6, 15].

## Material and Methods

Information on cell culture conditions, transfections and plasmids used in this study, on cell fractionation, mouse hippocampus dissection, culture of neurons and viral transduction, RNA immunoprecipitation (RIP), RNA isolation, reverse transcription and quantitative real-time PCR (qRT-PCR) and luciferase assays, as well as on miRNA analysis by Northern hybridization are found in the Supplementary Material and Methods.

### Mice

The animal welfare committees of the University of Freiburg and local authorities approved all mouse experiments, registered under the licence G14-096 or X14/04H. *Foxg1*^*cre/+*^ mice [17] were maintained in a C56BL/6 background. For experiments with wild-type (WT) mice, NMRI was used either at E13.5 or adult stages.

### SILAC and Mass Spectrometry

N2a cells were cultured in SILAC DMEM without arginine and lysine (#89985 ThermoScientific, Bremen, Germany) supplemented with Lys0/Arg0 or Lys8/Arg10 (0.398 mM L-arginine ^13^C_6_^15^N_4_, 0.798 mM L-lysine ^13^C_6_^15^N_2_, Euriso-Top, Saarbrücken, Germany) and 10% dialysed FCS (ThermoScientific). N2a cells were labelled for 12 passages with SILAC medium. Further processing is described in the Supplementary Material and Methods.

Analysis of the protein groups was done with the Perseus software [18]. Only proteins with two or more unique identified peptides, which were enriched more than 2-fold in both experiments, were considered. All raw data and original result files were deposited to the ProteomeXchange Consortium (http://proteomecentral.proteomexchange.org) via the PRIDE partner repository with the dataset identifier PXD007040.

### Immunoprecipitation

Immunoprecipitation (IP) was performed according to standard procedures as outlined in the Supplementary Material and Methods. For co-immunoprecipitations (co-IPs) with tagged FOXG1 (either FOXG1-Au1 or FOXG1-HA), mock conditions were either untransfected, empty vector or FOXG1 with the other tag. The following antibodies were used for IP: anti-Au1-tag (MMs-130R, Covance, Koblenz, Germany), anti-HA-tag (#3724, Cell Signaling), anti-DDX5 (rabbit, ab126730, abcam, Cambridge, UK), anti-FOXG1 (rabbit, ab18259, abcam) and anti-DROSHA (rabbit, ab12286, abcam).

### Immunoblotting

Immunoblotting was performed according to standard procedures as outlined in the Supplementary Material and Methods. The following antibodies were used for immunoblots: anti-Au1-tag (1:1000, MMs-130R, Covance), anti-HA-tag (1:1000, #3724, Cell Signaling), anti-DDX5 (1:2000, rabbit, ab126730, abcam), anti-FOXG1 (1:1000, rabbit, ab18259, abcam), anti-DROSHA (1:1000, rabbit, ab12286, abcam), anti-DGCR8 (1:1000, rabbit, ab191875, abcam), anti-H3 (1:1000, goat, ab12079, abcam), anti-NPM1 (1:1000, mouse, ab10530, abcam), anti-PRKAR2B (1:1000, DAKO) and anti-GAPDH (1:3000, ab8245, abcam). Densitometric analyses were done with ImageJ.

### RNA-Seq and Small RNA-Seq

Total RNA was prepared from the hippocampus with RNeasy kits (Qiagen), including on-column DNAse digestion. Samples were depleted from rRNA using RiboZero Gold kit (Illumina) before sequencing. Quality of the RNA was assessed with QIAxcel (#9001941, Qiagen, Hilden, Germany). Samples were prepared and analysed with Illumina HiSeq2500 (paired end, multiplexing run, 75 Mio/reads per sample). Bioinformatics analysis was performed using the Freiburger Galaxy Server [19, 20] as described in the Supplementary Material and Methods. For small RNA-Seq, 6-week-old *Foxg1*^*cre/+*^ mice hippocampi were used (*n* = 9). Raw data were deposited at the GEO database under the following accession numbers: small-RNAseq: GSE104169, mir-200 OE RNA-Seq: GSE106802 and *Foxg1*^*cre/+*^ hippocampus RNA-Seq: GSE106801.

### Proximity Ligation Assay

Proximity ligation assay (PLA) was performed with the Duolink starter kit reagents (DUO92103, SIGMA) according to the manufacturer’s instruction as outlined in the Supplementary Material and Methods. The following antibodies were used: anti-Au1 (1:2000, mouse, Covance) and anti-DDX5 (1:200, goat, ab10261, abcam) for PLA and anti-Lamin B1 (1:200, rabbit, ab133741, abcam). Images were taken with a confocal microscope and analysed with the LASX software (SP8, Leica, Jena, Germany).

### Statistical Analysis

GraphPad Prism software was used for statistical analyses. Statistical tests are indicated in the respective figure legends. Values in bar charts are expressed as average ± SEM. In in vivo experiments, each independent *N* is a different animal, and in in vitro experiments, each *N* is a different passage of cells. One sample Student’s *t* test was performed (e.g. on ΔΔCt values of qRT-PCRs) if measured variables could be paired (e.g. control and treatment of the same passage of cells). Unpaired Student’s *t* test (equal variances) or Welch’s *t* test (unequal variances) was used if variables were not paired (using in these cases for example ΔCt values for each sample group).

Final figures were prepared using FIJI (ImageJ, v. 2.0.0-rc-43/1.51d [21]) and Inkscape (v. 0.91).

## Results

### *Foxg1*^*cre/+*^ Animals Express Reduced Levels of Mature and Precursor miR200b/a/429 in the Hippocampus

**Fig. 1:**
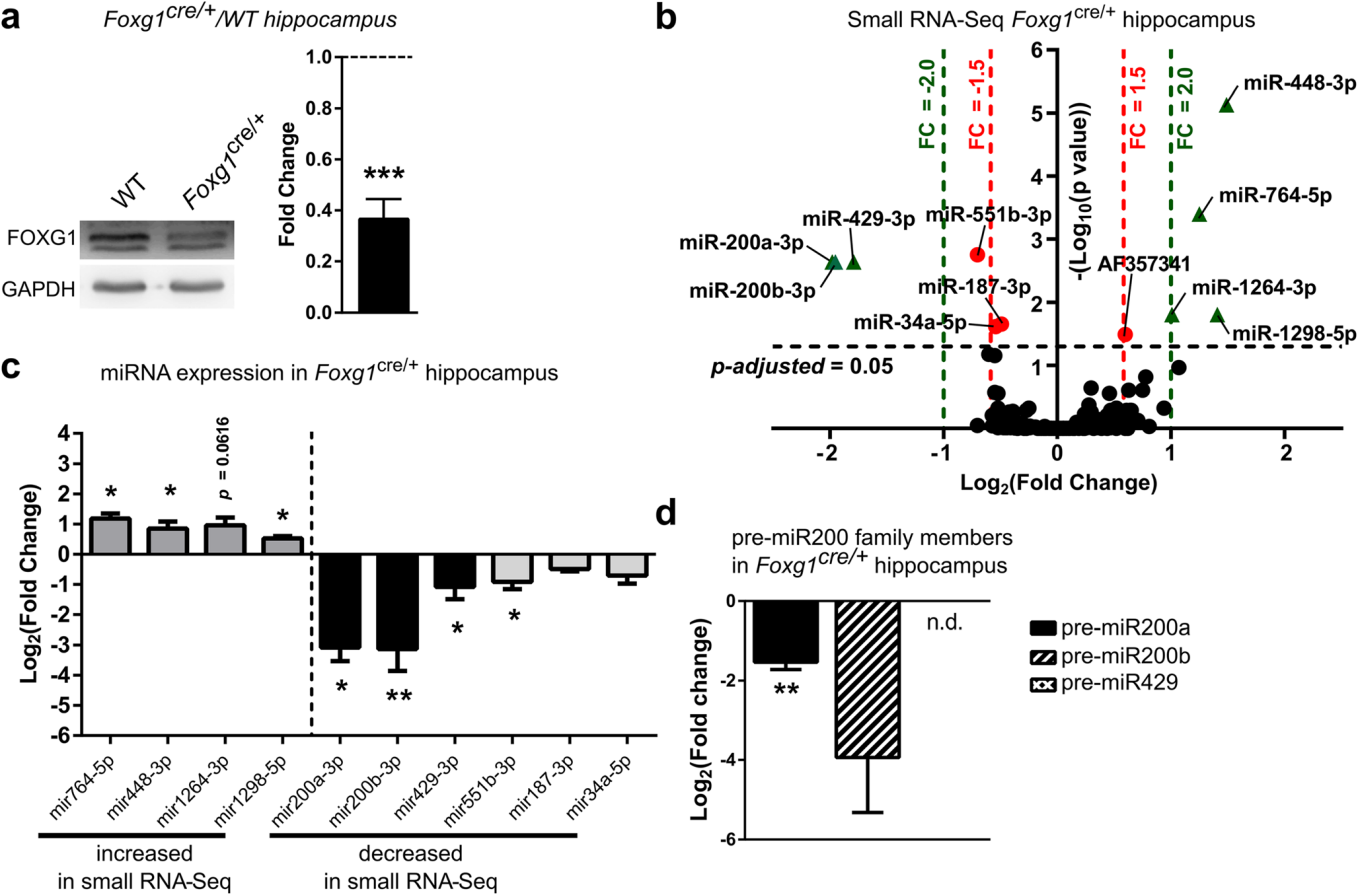
*Foxg1*^cre/+^ adult hippocampus expresses altered miRNA levels of miR200b/a/429 and *Htr2c* families. **a** Representative immunoblot and quantification of FOXG1 protein levels in *Foxg1*^cre/+^ and WT hippocampus show that FOXG1 protein is reduced by 60% in *Foxg1*^cre/+^ compared to control levels (dashed line). Mean with SEM, ***p < 0.001, one-sample Student’s *t* test. *n* = 6. **b** Volcano plot of small RNA-Seq of *Foxg1*^cre/+^ compared to WT hippocampus indicates that expression of miR200b/a/429 family is significantly decreased, whereas miRNAs from *Htr2c* gene significantly increased (dashed lines: DEseq2 *p*-adjusted value = 0.05 (black); FC = ± 2.0 (green); FC = ± 1.5 (red)). Values for adjusted *p* value were plotted on the *y*-axis. *n* = 9. **c** qRT-PCR validation confirms significantly altered expression levels of miRNAs of miR200b/a/429 and *Htr2c* families in 6-week-old hippocampus of *Foxg1*^cre/+^ animals. Mean with SEM, *p < 0.05, **p < 0.01, unpaired Student’s *t* test. *n* = 3. **d** Expression of precursor transcripts of miR200b/a/429 in *Foxg1*^cre/+^ hippocampus using qRT-PCR reveals decreased expression of pre-miR200b and pre-miR200a, while expression of pre-miR429 was not detectable (n.d.). Mean with SEM, *p < 0.05, **p < 0.01, unpaired Student’s *t* test. *n* = 3

As altered expression of miRNAs has been identified in tRTT [14, 15], we aimed to determine if miRNA expression was altered in a FOXG1 syndrome mouse model. We used 6-week-old *Foxg1*^*cre/+*^ mice, which expressed approximately half the amount of FOXG1 protein in the hippocampus compared to WT littermates (Fig. 1a) and performed small RNA sequencing (RNA-Seq) with hippocampi of *Foxg1*^*cre/+*^ and WT mice. This experiment revealed in total 11 small RNAs, including ten miRNAs, which were significantly altered with a fold change of at least ± 1.5 (Fig. 1b). Altered expression levels of these ten miRNAs were validated by qRT-PCR (Fig. 1c). Seven miRNAs, namely miR200a, miR200b, miR429, miR448, miR764, miR1264 and miR1298, had more than 2-fold and significantly altered expression levels in hippocampi of *Foxg1*^*cre/+*^ mice (Fig. 1b). Out of these, miR200b/a/429, which were decreased in *Foxg1*^*cre/+*^ hippocampus, derived from a single transcript (*Gm13648*)*.* miR448/764/1264/1298, which were increased in *Foxg1*^*cre/+*^ hippocampi, derived from the 5-hydroxytryptamine receptor 2C (*Htr2c*) transcript. Several reports suggested that miR200 family members control similar processes as FOXG1 in the developing cerebral cortex [5, 9, 22–28]. However, the functions of miR448/764/1264/1298 from the *Htr2c* gene are not known yet. We therefore decided to study the influence of FOXG1 on miR200 in more detail and performed qRT-PCR for precursor (pre-) miRNAs in 6-week-old male *Foxg1*^*cre/+*^ mice hippocampi. Pre-miR200a transcripts were significantly reduced in the hippocampus of the *Foxg1*^*cre/+*^ mice, whereas reduced levels of pre-miR200b did not reach significance and pre-miR429 was not detected (Fig. 1d). Expression of the primary transcript was neither detected by RNA-Seq nor by qRT-PCR in vivo (data not shown), suggesting very low expression levels and/or high turnover, e.g. by co-transcriptional processing [29]. Together, these data indicated that reduced levels of FOXG1 occurred alongside with reduced levels of pre-miR200a as well as mature miRNA 200b/a/429 levels in the adult hippocampus.

### *Prkar2b* Is a Target of FOXG1 and miR200 Family

**Fig. 2:**
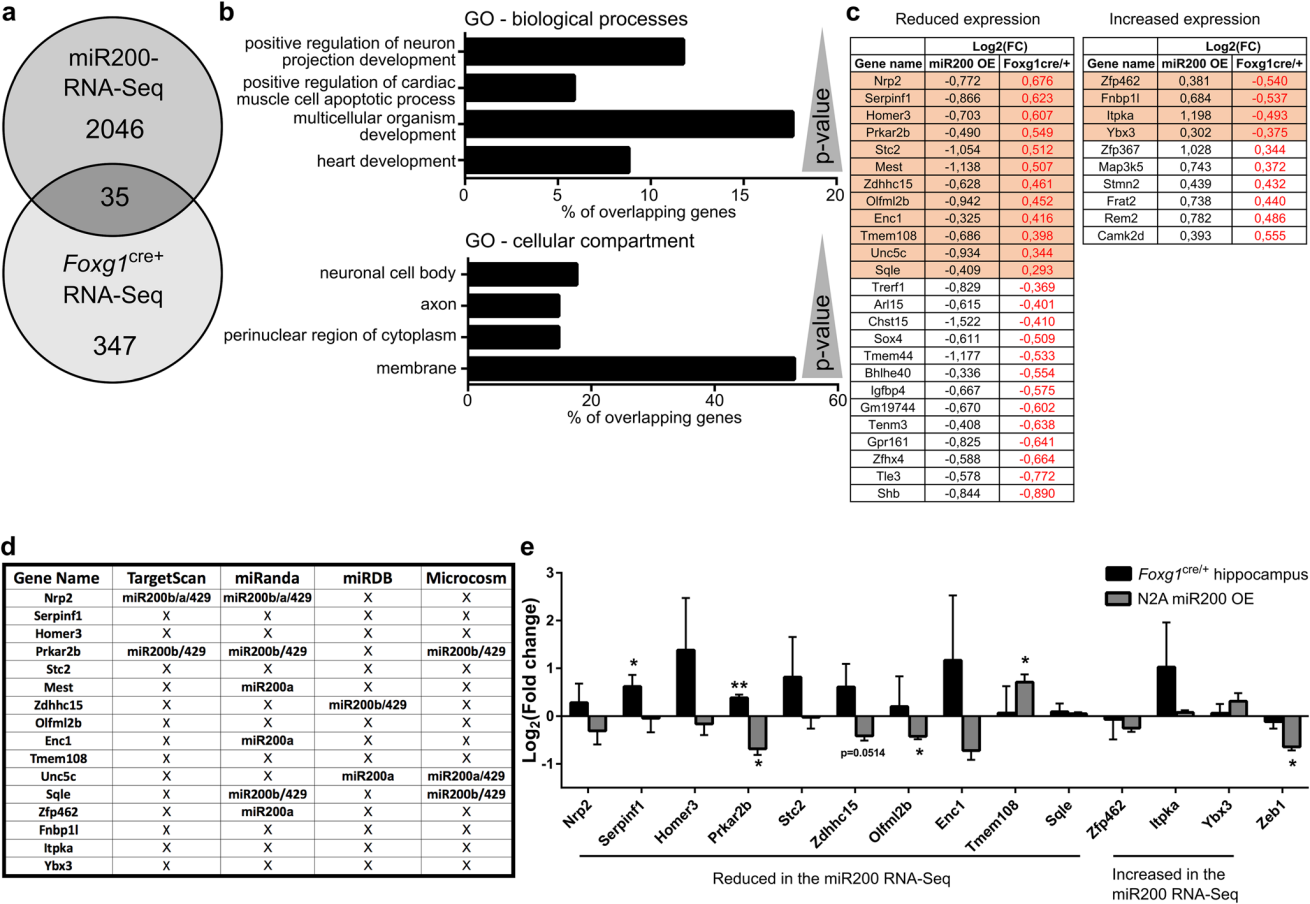
RNA-Seq after miR200 overexpression and of *Foxg1*^cre/+^ hippocampus identify *Prkar2b* as a miR200 target in the hippocampus. **a** Venn diagram depicting the overlap of 35 differentially expressed genes from RNA-Seq of N2a cells overexpressing miR200 family and from RNA-Seq of *Foxg1*^cre/+^ hippocampus. *n* = 2 and *n* = 3, respectively. **b** Bar chart of a DAVID GO term analysis for biological processes and cellular compartments of the 35 overlapping genes displayed in **a**. *p* values (as reported by DAVID) are given in the range of 0.0016 to 0.075. **c** Thirty-five overlapping genes with reduced or increased expression after miRNA200 family overexpression. Given is the log_2_(fold change) of both RNA-Seq datasets. Highlighted are genes, which show opposing expression level changes after miR200 family overexpression and in condition of less FOXG1 expression in *Foxg1*^*cre/+*^ hippocampus. **d** Table showing which of the 16 genes highlighted in **c** might be putative targets of miR200 family using four different prediction algorithms. Three out of four prediction algorithms identify miR200b and miR429 seed sequences in *Prkar2b*. **e** qRT-PCR validation of putative miR200 family target genes from **c**, together with *Zeb1*, which served as control for miR200 overexpression. miR200 family overexpression in N2a decreases *Prkar2b* levels compared to untransfected N2a cells, which are in turn increased in *Foxg1*^*cre/+*^ hippocampus with reduced levels of miR200 family member expression compared to wild type. Mean with SEM, *p < 0.05, **p < 0.01, Student’s *t* test (hippocampus samples) and one-sample Student’s *t* test (miR200 overexpression in N2a cells). *n* = 3–4

To identify miR200 targets with a putative role in FOXG1 syndrome, we performed RNA-Seq after overexpressing miR200 family members in N2a cells and compared it with RNA-Seq data obtained from adult *Foxg1*^*cre/+*^ hippocampus. In total, we identified 2081 differentially expressed genes after overexpressing miR200 family members and 382 genes in *Foxg1*^*cre/+*^ hippocampus. Intersection of the two RNA-Seq datasets revealed 35 genes shared between both datasets (Fig. 2a). The intersection of two independent datasets within a finite population size of 43,629 genes can be modelled as a hypergeometric distribution. We performed a hypergeometric test, which revealed a *p* value of 0.0001 that suggested that the overlap of 35 out of 382 *Foxg1*^*cre/+*^ and 2081 miR200 differentially expressed genes is not by chance but rather significant.

Gene ontology (GO) term analysis revealed that this set of 35 genes is classified to processes like neuronal projection and development (Fig. 2b). As the *Foxg1*^*cre/+*^ hippocampus expressed less mature miR200b/a/429, we focused on genes with opposing expression levels after miR200 overexpression compared to *Foxg1*^*cre/+*^ RNA-Seq to identify putative targets. Twelve of these genes showed reduced levels after miR200 overexpression, whereas four increased in expression (highlighted in Fig. 2c). We used different target prediction tools to analyse miR200 seed sequences on the mRNAs of these 16 genes. Three of the four prediction algorithms identified putative miR200 binding sites in the 3′-untranslated region (UTR) of *Prkar2b* (Fig. 2d). We subsequently assessed altered expression of 13 candidates by qRT-PCR in vitro and in vivo. *Prkar2b* mRNA levels decreased upon overexpression of the miR200 family members and increased in *Foxg1*^*cre/+*^ hippocampus (Fig. 2e) as predicted. Other candidates, i.e. *Serpinf1*, *Olfml2b* and *Tmem108*, were either significantly altered in only one condition or were altered in an opposing direction compared to the RNA-Seq data. These expression analyses therefore rendered *Prkar2b* as the best candidate for altered expression through FOXG1 and/or miR200 family.

**Fig. 3:**
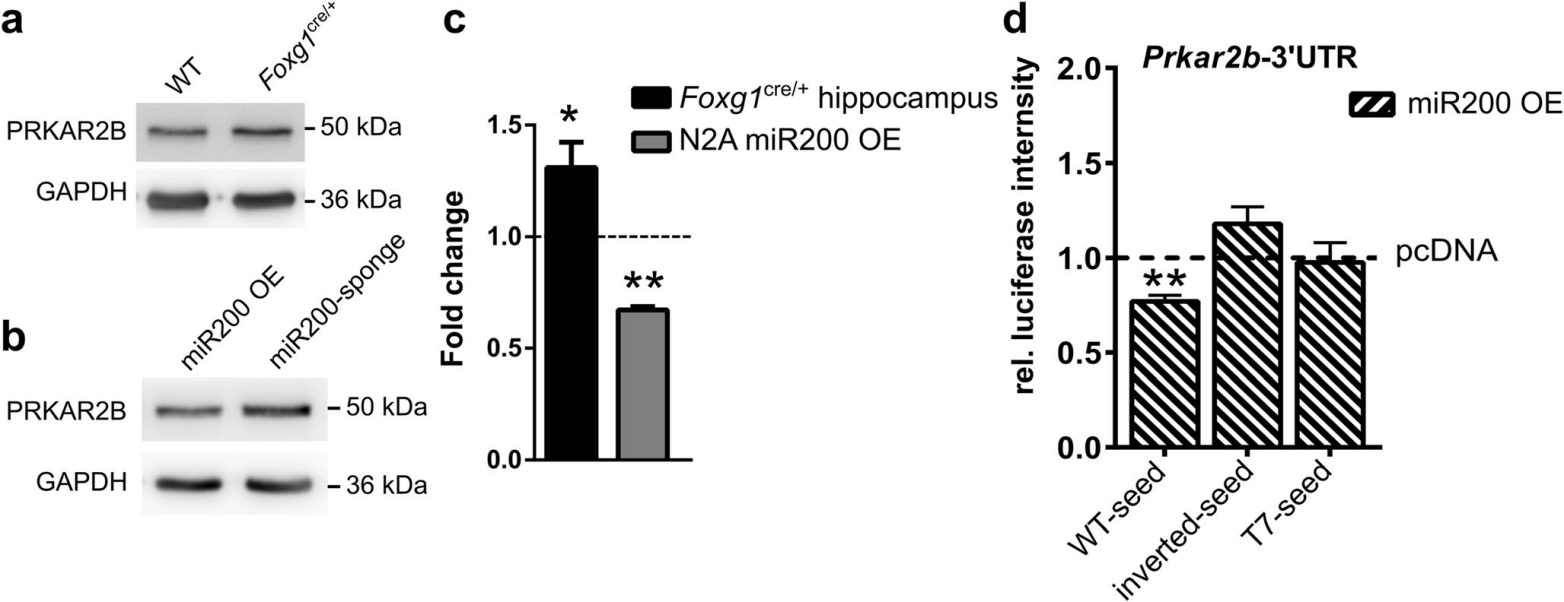
miR200b/a/429 overexpression reduces PRKAR2B protein levels. **a** Immunoblots of WT and *Foxg1*^*cre/+*^ hippocampus using anti-PRKAR2B antibodies show increased expression in the adult hippocampus of *Foxg1*^*cre/+*^ animals. *n* = 7. **b** Immunoblots of N2a cells overexpressing miR200 family or miR200 sponge plasmids. Overexpression of miR200 family reduces PRKAR2B levels compared to miR200 sponge-transfected N2a cells. *n* = 3. **c** Densitometric quantification of **a** and **b**. Reduced levels of FOXG1 result in increased levels of PRKAR2B in the adult hippocampus. miR200 family member overexpression reduces PRKAR2B levels significantly when compared to miR200 sponge (dashed line represents control expression levels). Mean with SEM, *p < 0.05, **p < 0.01, one-sample Student’s *t* test. *n* = 7. *Foxg1*^*cre/+*^ hippocampus, and *n* = 3 for miR200 OE. **d** Results of luciferase assays to detect degradation of a transcript containing a wild type (WT), inverted or T_7_ seed sequence in the *Prakr2b*-3′UTR. Overexpression of miR200b/a/429 degrades transcripts with WT-seed but not with inverted or T_7_ seed in the *Prkar2b*-3′UTR, when compared to control vector expression (dashed line). Mean with SEM, **p < 0.01, one-sample Student’s *t* test. *n* = 3–5

Next, we analysed altered protein levels of PRKAR2B in the *Foxg1*^*cre/+*^ hippocampus using immunoblotting. As anticipated, protein levels of PRKAR2B increased with reduced levels of FOXG1 in vivo (Fig. 3a, c) and decreased after overexpression of miR200 compared to a miR200 sponge in N2a cells (Fig. 3b, c). To further show that the *Prkar2b* 3′UTR was targeted by miR200b/a/429, we used a luciferase reporter assay. Overexpression of miR200b/a/429 together with a plasmid carrying luciferase followed by the WT 3′UTR of *Prkar2b* reduced the luciferase signal. In contrast, inverted or T_7_ replaced seed sequences in the 3′UTR of *Prkar2b* did not affect luciferase expression (Fig. 3d). We therefore concluded that *Prkar2b* was a direct target of miR200 family members.

**Fig. 4:**
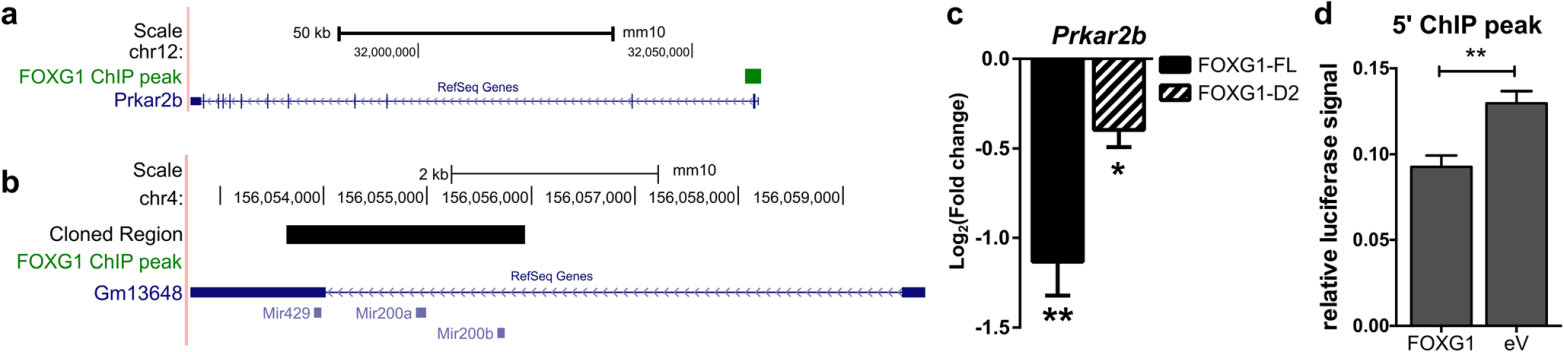
FOXG1 suppresses *Prkar2b* expression through direct and indirect mechanisms. **a** UCSC Genome browser view of *Prkar2b* gene region indicating FOXG1 binding site (FOXG1 ChIP peak, shown in green) from FOXG1 adult hippocampus ChIP-Seq data. **b** UCSC genome browser view of miR200b/a/429 gene showing the cloned region in the black bar, which does not include promoter, 5′ and 3′UTR regions. No FOXG1 binding sites were predicted in the miR200b/a/429 gene. **c** Transcript levels of *Prkar2b* are reduced after FOXG1-FL and FOXG1-D2 expression in N2a cells compared to empty vector-transfected cells. Mean with SEM, *p < 0.05, **p < 0.01. *n* = 5. **d** Results of luciferase assays to detect transcriptional influence of FOXG1 on the region around the peak enriched after FOXG1–ChIP-Seq in the 5′ region of the *Prkar2b* gene. FOXG1 suppresses luciferase activity significantly, when compared to control. Mean with SEM, **p < 0.01, unpaired Student’s *t* test. *n* = 5

As FOXG1 is described as a transcription factor, we analysed FOXG1 chromatin-immunoprecipitation sequencing (ChIP-Seq) from E14.5 cortical tissue (available through the Active Motif web site) and own ChIP-Seq data from adult hippocampus (data not shown) for enriched genomic regions around the *Prkar2b* and *Gm13648* genes. We identified a peak at the 5′ end of *Prkar2b*, whereas no enrichment was observed in *Gm13648* (Fig. 4a, b). We therefore analysed whether FOXG1 suppressed *Prkar2b* expression through regulative sequences at the 5′ end. First, overexpression of full-length FOXG1 in N2a cells resulted in decreased transcription of *Prkar2b*. Interestingly, overexpression of a Forkhead box-deficient variant of FOXG1 also led to a reduction, albeit smaller than in the presence of full-length FOXG1 (Fig. 4c). We next used a luciferase assay to verify putative regulative sequences within the 5′ end of *Prkar2b*. We cloned the 5′ region of the *Prkar2b* gene containing a predicted Forkhead box binding site (Fig. 4a) upstream of a luciferase reporter. In this assay, overexpression of FOXG1 reduced luciferase activity compared to the empty vector control (Fig. 4d). These results indicated that FOXG1 suppressed *Prkar2b* transcription directly in addition to its degradation activities via miR200 family members. Together, the data provided strong evidence that PRKAR2B is a novel candidate protein, misexpression of which might be implicated in FOXG1 syndrome.

To investigate if levels of miR200 family members or *Prkar2b* were altered in other regions than the hippocampus of *Foxg1*^*cre/+*^ animals, we assessed the respective transcript levels in samples derived from the cerebral cortex and olfactory bulb. Neither mature miR200b/a/429 nor *Prkar2b* levels changed in the cerebral cortex or olfactory bulb in *Foxg1*^*cre/+*^ compared to WT animals (Fig. S1a–c).

### FOXG1 Interacts with DDX5 and DROSHA Microprocessor Complex

**Fig. 5:**
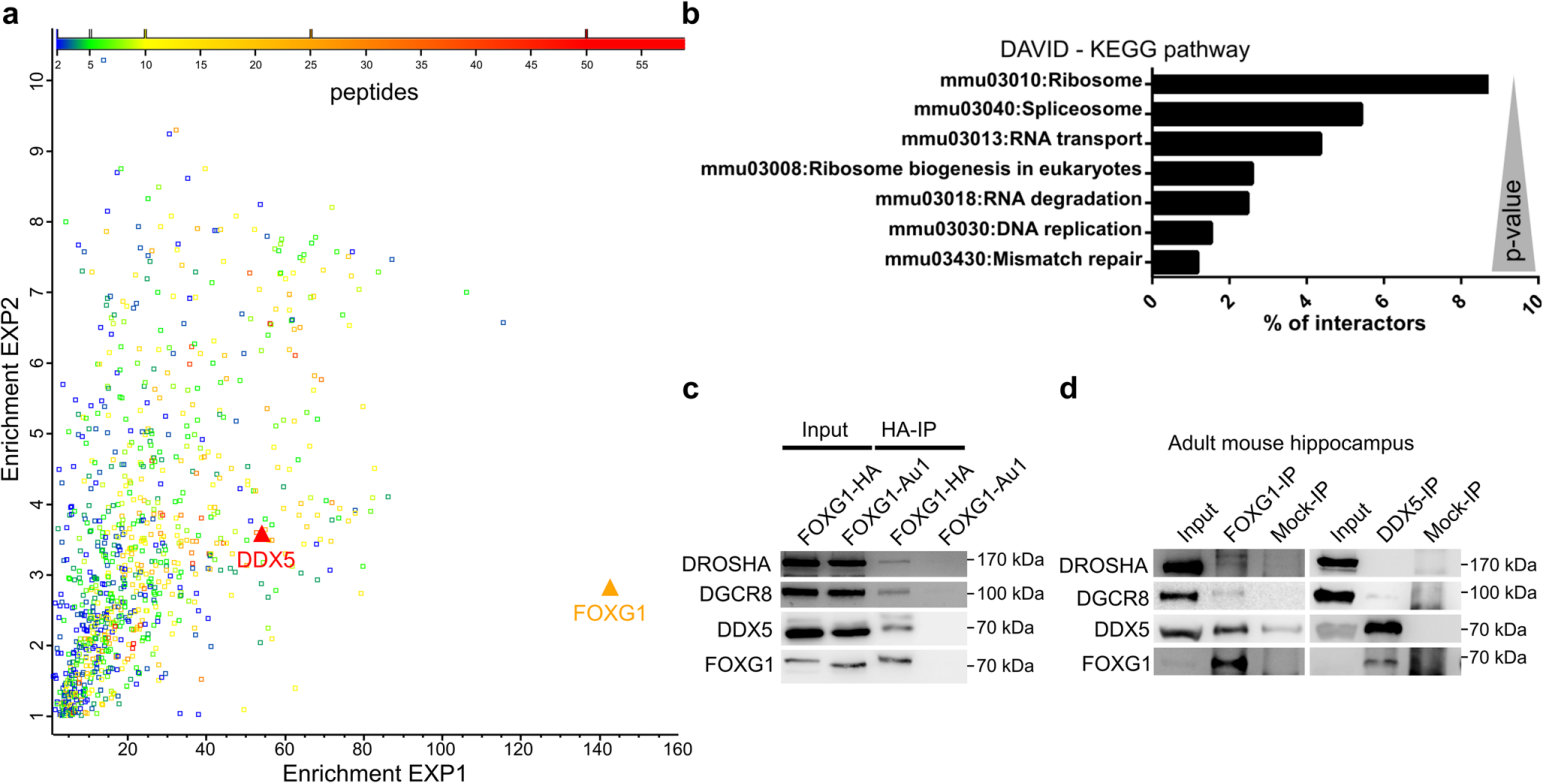
Mass spectrometry and Co-IP reveal interaction of FOXG1 with DDX5 and the microprocessor. **a** Scatterplot of the FOXG1 interactome highlights its interaction with DDX5. The fold enrichment of two independent FOXG1-Au1 co-IP replicates of the MS analysis are plotted on the *x*- and *y*-axis, respectively, and detected proteins are colour coded with regard to the number of individual peptides mapping into the identified protein. *n* = 2. **b** DAVID KEGG pathway analysis with the most significant pathways. *p* values < 0.049. **c** Representative immunoblot after FOXG1-HA co-IP using anti-HA, anti-DDX5, anti-DROSHA and anti-DGCR8 shows that DDX5 and the microprocessor proteins interact with FOXG1 in N2a cells. Overexpression of FOXG1-Au1 serves as control for specificity of the HA-co-IP. **d** Representative immunoblot using anti-FOXG1, anti-DDX5, anti-DROSHA and anti-DGCR8 antibodies after co-IP of endogenous FOXG1 and DDX5, respectively, from protein extracts of adult mouse hippocampus. *n* = 3

As our data indicated that FOXG1 was not directly involved in transcriptional control of the pri-miR200 transcript, we aimed to identify protein interaction partners of FOXG1 that might explain the posttranscriptional effects observed. We overexpressed FOXG1 in SILAC-labelled N2a cells and performed FOXG1 co-IP followed by quantitative mass spectrometry (MS). MS analysis identified 701 proteins with at least two unique identified peptides, which were enriched more than 2-fold by FOXG1 co-IP (Fig. 5a). We analysed the MS dataset using *Database for Annotation, Visualization and Integrated Discovery* (DAVID) which revealed *Kyoto Encyclopedia of Genes and Genomes* (KEGG) pathway terms related to RNA metabolism, i.e. spliceosome, RNA transport and RNA degradation (Fig. 5b). These data strongly suggested that FOXG1 might be involved in RNA metabolism in addition to its transcriptional repressor activity. The spliceosome pathway had a *p* value of 8.71E-25, and one of the strongest enriched proteins overall and among RNA binding proteins was the ATP-dependent RNA helicase DDX5 (Fig. 5a). DDX5 regulates posttranscriptional control of gene expression at various levels [30]. Posttranscriptional control is affected in MECP2-mediated Rett syndrome [31] as well as in other autism spectrum disorders [32]. Therefore, we decided to focus on the interaction between FOXG1 and DDX5 to elucidate a novel function for FOXG1 in posttranscriptional RNA regulation. We confirmed this novel interaction between FOXG1 and DDX5 after overexpression of HA- or Au1-tagged FOXG1 in N2a cells (Fig. 5c), or with endogenous FOXG1 in adult mouse hippocampus (Fig. 5d) using co-IP followed by immunoblotting. FOXG1 interacted with DDX5 both in vitro and in vivo. Since DDX5 associates with the microprocessor complex [33] and has an important role in miRNA maturation [34], we probed FOXG1-co-IP samples for DROSHA and DiGeorge syndrome critical region gene 8 (DGCR8). FOXG1 interacted with both DROSHA and DGCR8 in vitro and in vivo (Fig. 5c, d), suggesting that FOXG1 may influence miRNA maturation.

### FOXG1–DDX5 Complex Interacts with the Microprocessor in the Nucleus

**Fig. 6:**
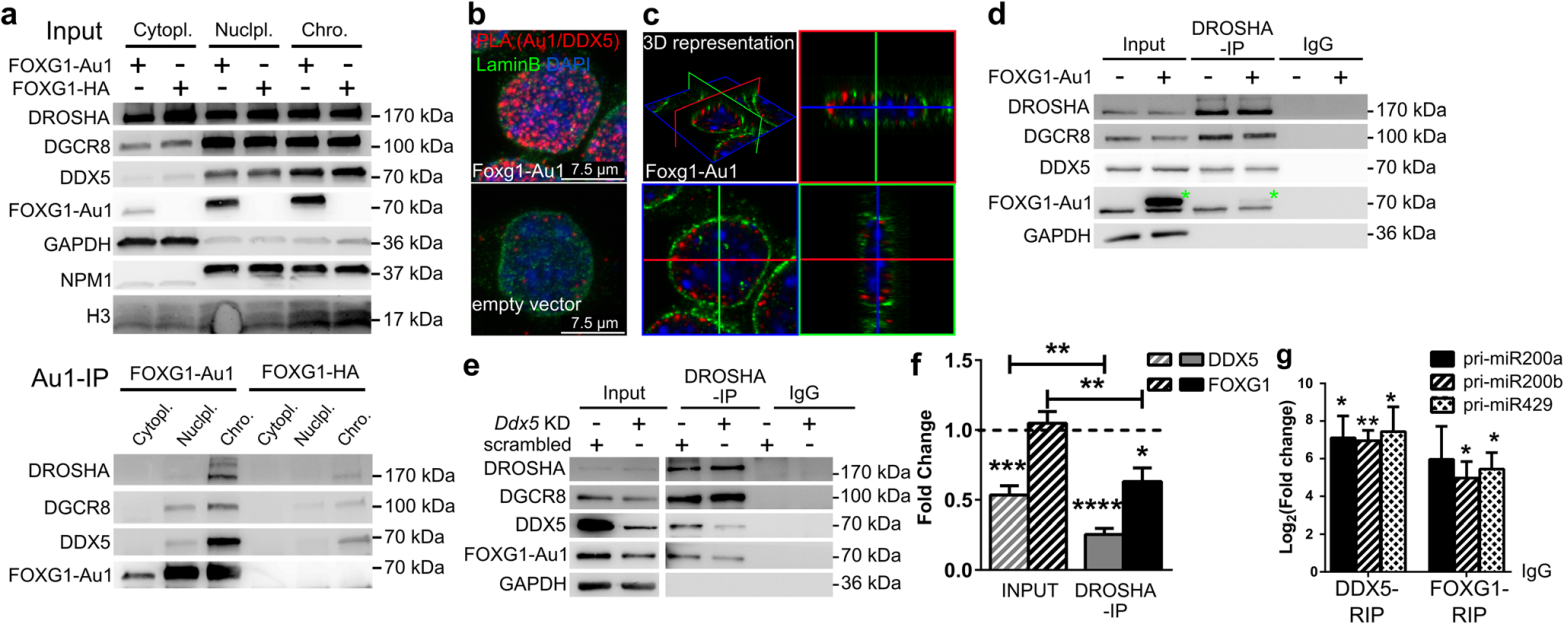
FOXG1 and DDX5 interact in the nucleus and DDX5 recruits FOXG1 to DROSHA. **a** Immunoblots of cytoplasmic (cytopl), nucleoplasmic (nuclpl) and chromatin (chro) fractions after cell fractionation of FOXG1-Au1 or FOXG1-HA expressing N2a cells using anti-Au1, anti-DDX5 and microprocessor antibodies (upper panel, input samples). GAPDH, NPM1 and H3 are used as controls for fractionation. Au1-co-IP of FOXG1-Au1 or FOXG1-HA expressing N2a cells and immunoblots with anti-Au1, anti-DDX5 and microprocessor proteins showing that interactions take place in the nucleoplasm and chromatin fraction (lower panel, Au1-IP samples). *n* = 3. b Confocal imaging of PLA of FOXG1-Au1 and DDX5 after FOXG1-Au1 overexpression in N2a cells shows that FOXG1/DDX5 localises near Lamin B-positive immunostaining inside the nucleus. Scale bar 7.5 μm. *n* = 2. **c** 3D representation of **b**. **d** Immunoblot after DROSHA co-IP of FOXG1-Au1 overexpressing or empty vector-transfected N2a cells using anti-Au1, anti-DDX5 and anti-microprocessor antibodies. DDX5 co-precipitates with DROSHA in the presence and absence of FOXG1. Green asterisks indicate FOXG1-Au1 band. *n* = 2. **e** Immunoblot of DROSHA co-IP after *Ddx5* KD or scrambled control transfection in FOXG1 overexpressing N2a cells. Antibodies as in **d**. DROSHA co-precipitates less FOXG1 in conditions of decreased DDX5 expression. *n* = 6. **f** Densitometric analysis of **e**. Input FOXG1 and DDX5 are normalised to GAPDH, and in DROSHA-IP, FOXG1 and DDX5 are normalised to DROSHA (dashed line). Ratios of *Ddx5* KD to scrambled control are represented. Additional comparison of FOXG1 and DDX5 levels between DROSHA-IP and input revealed statistical significant reduction of FOXG1 and DDX5 after *Ddx5* KD (represented by the straight horizontal line bars). Mean with SEM, *p < 0.05, **p < 0.01 ***p < 0.001, ****p < 0.0001, one-sample Student’s *t* test. *n* = 6. **g** qRT-PCR analyses of pri-miR200 family members after native DDX5 and FOXG1-Au1 RIP showing that FOXG1 and DDX5 co-precipitate pri-miR200 transcripts normalised to IgG control. Mean with SEM, *p < 0.05, **p < 0.01, unpaired Student’s *t* test. *n* = 3

The maturation process of miRNAs is spatially separated. The nuclear DROSHA microprocessor excises the pre-miRNA from the primary transcript, whereas subsequent cleavage of mature miRNAs in the form of the stem loop occurs through the cytoplasmic DICER complex. To identify the cellular localization of the FOXG1–DDX5 complex, we used co-IPs from fractionated cells. In N2a cells, DROSHA localised not exclusively to the nucleus but was detected at significant levels in the cytoplasm. Cytoplasmic localisation of DROSHA depends on phosphorylation [35, 36] and splicing [37]. Despite the enriched cytoplasmic localisation of DROSHA, we precipitated FOXG1 along with DDX5, DROSHA and DGCR8 from the nucleoplasmic and chromatin fraction (Fig. 6a). We used PLA and confirmed that the FOXG1–DDX5 complex localised to the nucleus in vivo in N2a cells (Fig. 6b). Confocal imaging and 3D stacking of FOXG1–DDX5 PLA in N2a cells revealed that the PLA signal of FOXG1–DDX5 localised to the proximity of the inner nuclear membrane (Fig. 6c). This result suggested that nuclear FOXG1–DDX5 complexes did not directly affect DICER-mediated processing, as their interaction was restricted to the nuclear compartment.

We next investigated if FOXG1 mediated the binding of DDX5 to the microprocessor in neural cells, or vice versa. We performed DROSHA co-IP after overexpressing FOXG1 in N2a cells and assessed DDX5 levels. DROSHA co-precipitated similar levels of DDX5, irrespective of the presence of FOXG1 (Fig. 6d). Next, we investigated if knockdown (KD) of DDX5 affected FOXG1 binding to DROSHA. By reducing expression of DDX5, we observed decreased FOXG1 recruitment to DROSHA (Fig. 6e, f). Together, these data suggested that DDX5 associated to the microprocessor independent of the presence of FOXG1, but that FOXG1 required DDX5 to bind to DROSHA.

FOXG1 did not seem to alter pri-miR200b/a/429 transcription and it associated with the microprocessor in the nucleus. We therefore hypothesised that a FOXG1–DDX5 complex might affect miR200 biogenesis and assessed whether FOXG1 or DDX5 would bind the pri-miR200b/a/429 transcript. We applied native RIP with antibodies against the Au1-tag after FOXG1-Au1 overexpression in N2a cells, or with anti-DDX5, respectively. RIP was followed by qRT-PCR, which revealed that both FOXG1 and DDX5 precipitated the pri-miR200b/a/429 (Fig. 6g).

### Decreased Levels of DDX5 in FOXG1 Overexpressing Cells Reduce Mature miR200 Levels but Increase DROSHA Processivity

**Fig. 7:**
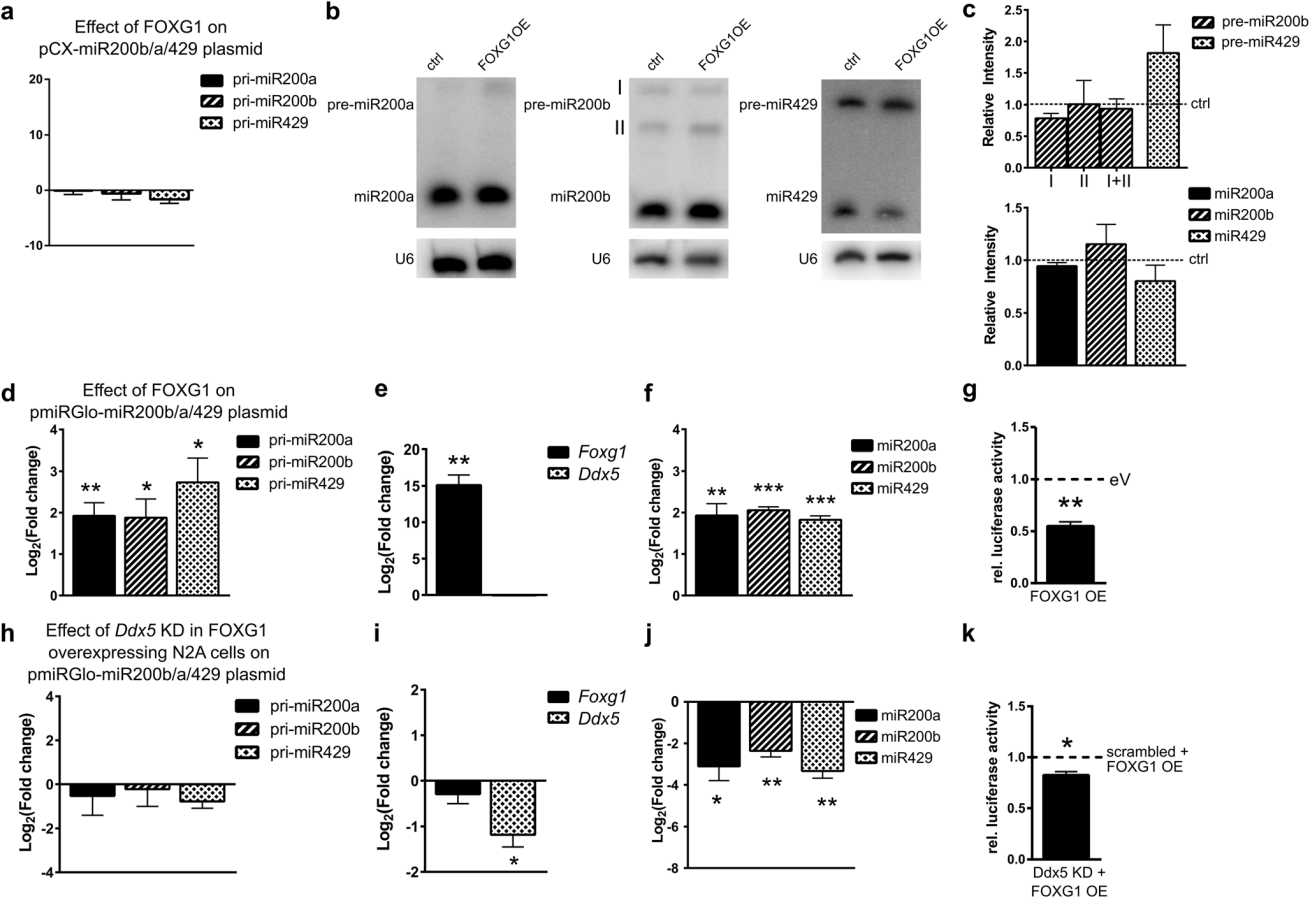
FOXG1 overexpression affects miR200 family levels in conditions of increased expression of pri-miR200 in a DDX5-dependent manner. **a** qRT-PCRs of pri-miR200b/a/429 of N2a cells overexpressing FOXG1 and miR200 family using pCX-miR200b/a/429 compared to an empty vector. One-sample Student’s *t* test. *n* = 3. **b** Representative northern blots of precursor (pre-) and mature miR200b/a/429 in N2a cells using pCX-miR200b/a/429. Shown are control-transfected and FOXG1 overexpression in N2a cells probed for *pre/miR200a* (upper panel), *pre-miR200b* (middle panel) and pre-miR429 (lower panel) as well as U6 loading control. **c** Densitometric quantification of the northern blot bands for precursors of miR200b (isoforms I and II) and miR429 (left panels) and mature miR200b/a/429 (right panels). Dashed lines indicate expression levels of control cells. FOXG1 overexpression does not alter expression levels of precursor and mature miR200 family members. Mean with SEM, **p < 0.01, one-sample Student’s *t* test. *n* = 3. **d** qRT-PCRs of pri-miR200b/a/429 of N2a cells overexpressing miR200 family using pmiRGlo-miR200b/a/429 and FOXG1 compared to empty vector. pri-miR200 levels increase upon FOXG1 overexpression. Mean with SEM, *p < 0.05, **p < 0.01, one-sample Student’s *t* test. *n* = 3–6. **e** qRT-PCR results confirm increased *Foxg1* and unchanged *Ddx5* expression. Mean with SEM, **p < 0.01, one-sample Student’s *t* test. *n* = 4. **f** qRT-PCRs of mature miR200b/a/429 of N2a cells overexpressing miR200 family from pmiRGlo-miR200b/a/429 plasmid and FOXG1 compared to empty vector expression. Levels of mature miR200 increase statistically significant. Mean with SEM, **p < 0.01, ***p < 0.001, one-sample Student’s *t* test. *n* = 5–6. **g** Reduced luciferase activity indicates an increased turnover of pri-miR200b/a/429 in the presence of FOXG1. Mean with SEM, **p < 0.01, one-sample Student’s *t* test. *n* = 4. **h** qRT-PCRs of pri-miR200b/a/429 of N2a cells overexpressing miR200 family using pmiRGlo-miR200b/a/429 and FOXG1 as well as simultaneous *Ddx5* KD, compared to FOXG1 overexpression and scrambled control. pri-miR200 levels are unaffected by *Ddx5* KD. Mean with SEM, one-sample Student’s *t* test. *n* = 3–6. *i* qRT-PCR results confirm decreased *Ddx5* levels after *Ddx5* KD and unchanged *Foxg1* expression. Mean with SEM, *p < 0.05, one-sample Student’s *t* test. *n* = 4. **j** qRT-PCRs of mature miR200b/a/429 in N2a cells after overexpressing miR200 family from pmiRGlo-miR200b/a/429 plasmid and FOXG1 as well as simultaneous *Ddx5* KD compared to FOXG1 overexpression and scrambled control. Levels of mature miR200 decrease significantly after reduction of DDX5 in FOXG1 expressing cells. Mean with SEM, *p < 0.05, **p < 0.01, one-sample Student’s *t* test. *n* = 5–6. **k** Reduced luciferase activity indicates an increased turnover of pri-miR200b/a/429 after *Ddx5* KD and FOXG1 overexpression when compared to cells overexpressing FOXG1 and scrambled control. Mean with SEM, *p < 0.05, one-sample Student’s *t* test. *n* = 4

We next aimed to elucidate the molecular level at which FOXG1 and DDX5 affected miR200 biogenesis. First, we assessed whether overexpression of FOXG1 alone would be sufficient to affect miR200 biogenesis. Using pCX-miR200b/a/429 plasmid to express miR200b/a/429 family in N2a cells did not increase the expression of the primary transcript after FOXG1 overexpression (Fig. 7a), confirming that FOXG1 did not influence transcription of the parental gene. We next determined the levels of pre-miR200 and mature miR200 after FOXG1 and miR200b/a/429 family overexpression using Northern blots. Overexpression of FOXG1 in these conditions did neither affect precursor nor mature miR200 levels (Fig. 7b, c).

We next used a pmiRGLO-miR200b/a/429 plasmid to overexpress the miR200b/a/429 family and assessed whether levels of primary miR200 transcripts changed after FOXG1 overexpression. In these conditions, the presence of FOXG1 significantly increased pri-miR200b/a/429 levels (Fig. 7d, e), which resulted in significantly increased levels of mature miR200 family expression (Fig. 7f). Moreover, the N2a cells transfected with pmiRGlo-miR200b/a/429 along with FOXG1 allowed us to observe an increased turnover of the pri-miR200b/a/429 in a luciferase reporter assay (Fig. 7g). As we observed altered mature miR200 levels only when we used pmiRGLO-miR200b/a/429, which led to increased levels of the primary transcript, but not with the pCX-miR200b/a/429 plasmid that did not change pri-miR200 levels, we concluded that FOXG1 alone is probably not sufficient to affect miR200 biogenesis. Instead, significantly altered levels of the primary transcript are necessary to observe altered expression of mature miR200 family members.

To further address whether DDX5 would play a role in the FOXG1-mediated increase in mature miR200 levels as observed in Fig. 7f, we knocked-down (KD) *Ddx5* in FOXG1 and pri-miR200b/a/429 (using pmiRGLO-miR200b/a/429) expressing cells (Fig. 7i). Under these conditions, pri-miR200b/a/429 levels did not change (Fig. 7h); however, the levels of the mature miR200 family decreased significantly (Fig. 7j), despite increased activity of the microprocessor (Fig. 7k). These findings indicated that DDX5 is involved in FOXG1-mediated processing of the miR200 family members.

**Fig. 8:**
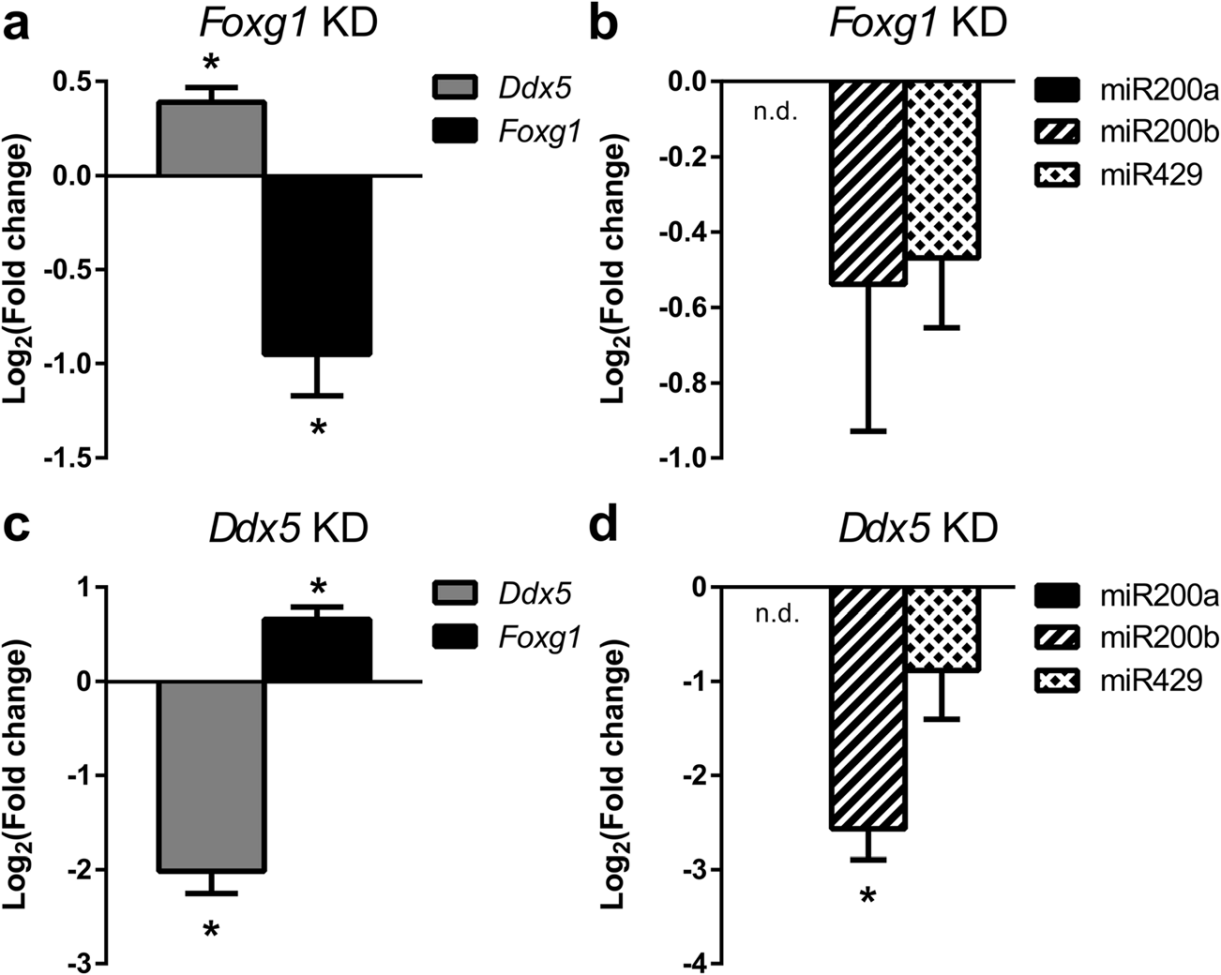
Increased levels of *Foxg1* and *Ddx5* KD decrease miR200b expression in primary hippocampal neurons. **a** qRT-PCR of *Ddx5* and *Foxg1* in *Foxg1* KD primary hippocampal cells, *Foxg1* KD increases expression of *Ddx5.***b** qRT-PCR of mature miR200 family expression after KD of *Foxg1*. Expression levels of mature miRNA200 family do not change. **c** qRT-PCR of *Ddx5* and *Foxg1* in *Ddx5* KD primary hippocampal cells. *Ddx5* KD results in increased expression of *Foxg1.***d** qRT-PCR of mature miR200 family expression after KD of *Ddx5*. Reduced expression of *Ddx5* with concomitant increase in *Foxg1* expression causes significant reduction of mature miR200b expression in primary hippocampal neurons. All data are represented as mean with SEM, *p < 0.05, **p < 0.01, one-sample Student’s *t* test. *n* = 3

As the experimental conditions in N2a cells required overexpression of FOXG1 and increased levels of pri-miR200b/a/429, we aimed to study the influence of FOXG1 and DDX5 in primary hippocampal neurons. KD of FOXG1 resulted in increased levels of DDX5, without significant effect on miR200 maturation (Fig. 8a, b). In contrast, KD of DDX5 resulted in concomitant increase of FOXG1 expression (Fig. 8c), as well as significantly decreased expression of miR200b (Fig. 8d). This result mimicked and confirmed our observation in N2a cells, in which decreased levels of DDX5 and concomitant increased levels of FOXG1 affected miR200 maturation. Taken together, these data showed that FOXG1 and DDX5 were involved in miR200 biogenesis and that the underlying molecular mechanism depended on a crucial balance between the levels of FOXG1, DDX5 and pri-miR200b/a/429.

## Discussion

In this study, we identified that FOXG1 affects PRKAR2B expression at multiple levels, on one hand through direct transcriptional repression and on the other hand through the miR200 family that directly targets *Prkar2b* transcripts. Thereby, we discovered important new roles of FOXG1 for the function of neural cells and of hippocampal neurons in the adult CNS, independent on proliferative effects (Fig. S2). Patients with mutations in *FOXG1* show impaired neuronal function and the symptoms are comparable to Rett syndrome. However, the role of FOXG1 in mature neurons and in the adult CNS is not entirely clear. In the adult hippocampus, FOXG1 prevents depletion of the progenitor pool in the dentate gyrus [38]. In cerebellar granule neurons, FOXG1 promotes survival [39]. Also, FOXG1 binds to a spliced, proapoptotic version of MECP2 thereby preventing neuronal death [13].

Apart from influencing gene transcription, Pancrazi et al. showed that FOXG1 localised within the mitochondrial matrix and regulates the mitochondrial membrane potential, mitochondrial fission and mitosis [40]. This finding gave indication that FOXG1 might have additional functions than regulation of transcription, for example in posttranscriptional control. Here, we describe a novel interaction between FOXG1, DDX5 and the microprocessor complex that can affect maturation of miR200b/a/429. Our data show that the PKA regulator PRKAR2B is a direct target of FOXG1 as well as of miR200 family. The biogenesis of miR200 can also be influenced by FOXG1, probably in a context-dependent manner that depends on the levels of the different players. Our data robustly show that increased levels of FOXG1 in *Ddx5* KD N2a and hippocampal cells decreased mature miR200. However, at least in N2a cells, we observed decreased miR200 levels despite a significantly increased turnover of the primary transcript. Therefore, we hypothesise further regulative layers downstream of DROSHA that might be affected by FOXG1. Our MS data propose different pathways that might act downstream of DROSHA, as the data suggest that FOXG1 associates for example with the exosome and therefore confers degradation of pre-miR200 family members. It is also possible that FOXG1 interacts with nuclear envelope proteins and affects the transport of pre-miR200 into the cytoplasm. The PLA signal suggests that FOXG1/DDX5 complexes localise near the membrane, which might corroborate such interpretation. However, to shed more light on FOXG1’s implication in posttranscriptional control and FOXG1/DDX5-mediated effects downstream of DROSHA, much more research is needed.

Our finding that FOXG1 influences posttranscriptional maturation of miRNAs reflects a shared cellular function between FOXG1 and MECP2, as the latter also associates to DROSHA and DGCR8 to regulate miRNA processing in the adult mouse hippocampus [41]. However, while the involvement of FOXG1 and MECP2 in miRNA biogenesis is conserved, targets are different. MECP2 suppresses miR134, miR383, miR382 and miR182 maturation in the hippocampus [41]. Our study revealed fewer numbers of differently expressed miRNAs that are affected in *Foxg1*^*cre/+*^ hippocampus compared to the data reported for MECP2 deficiency. And, we identified no commonly misregulated miRNAs between the two forms of RTT. Thus, although both MECP2 and FOXG1 associate with the microprocessor complex, they seemingly affect maturation of different miRNAs.

miR200b/a/429 transcription is regulated by different factors in a tissue-specific manner. TP53 is a transcription factor necessary for miR200b/a/429 gene expression [42, 43], whereas ZEB1 and ZEB2 repress expression of the miR200b/a/429 gene. Ovarian tumours induce miR200 expression upon DNA damage involving another RNA helicase, namely DDX1 [44]. Control of expression and biogenesis of the miR200 is necessary as this family is implicated in diverse cellular processes, ranging from neurodegeneration, eye development, adipocyte differentiation, taste bud and tooth development to maintenance of stem cell identity (reviewed in [45]). The miR200 family has important regulative functions in cancer [46] and neural differentiation in humans [28], zebrafish [26], *Drosophila* [47] and neural PC12 cells [27], as well as in mice [24, 25]. Altogether, miR200 controls similar processes as FOXG1 in the developing cerebral cortex [5, 9, 22, 23]. Interestingly, *Foxg1* has been proposed as miR200 target in different model systems [26, 48, 49], and varying FOXG1 expression levels might thus be regulated through a miR200-dependent feedback loop. Such feedback loop might account for the transiently reduced FOXG1 expression in differentiating progenitors in the cerebral cortex and the reinitiation of its expression in differentiated projection neurons [6]. It is therefore tempting to speculate further that the biogenesis of miR200, influenced by FOXG1, is not restricted to the adult hippocampus but also takes place in the developing cerebral cortex. This notion is supported by our identification of FOXG1–DDX5 interaction in E13.5 embryonic cortical tissue (data not shown).

Another important finding of our study is that FOXG1 and miR200 target PRKAR2B in the CNS. PRKAR2B is the target of miR200b in platelets [50]. PRKAR2B is a regulative type 2 subunit of protein kinase A (PKA), which is expressed in different tissues but has the highest expression in the CNS [51]. PKA signalling is critically implicated in memory formation, and this function is evolutionary highly conserved (reviewed by [52]). For example, PKA regulates synaptic plasticity by phosphorylating AMPA receptor subunits [53], or the GluN2B subunit of NMDAR during emotional response to stress [54]. Interference with PKA signalling in the mouse hippocampus impairs long-term spatial memory formation and elicits long-term memory deficits in contextual fear conditioning [55]. In the latter, freezing behaviour reduces after interference with PKA signalling. *Foxg1*^*cre/+*^ mice show a similar behaviour of decreased contextual fear response [56]. Thus, increasing levels of the repressive PRKAR2B subunit after decreased expression of miR200 family members might interfere with effective PKA signalling in *Foxg1*^*cre/+*^ mice. Strikingly, MECP2-deficient mice show the same decreased freezing behaviour [57], and increased levels of PRKAR2B (data not shown and [58]). Published data from iPS-derived neurons show that *PRKAR2B* expression levels are altered in some FOXG1 syndrome patients (log_2_FC of *PRKAR2B* of 1.14 with a FDR of 8E-04 [59]). In all, impaired PKA signalling might be a common feature in tRTT and atRTT. Altered levels of PRKAR2B might also be responsible for other phenotypic alterations in Rett syndrome, such as altered motor behaviour [60], or impaired vision [61–64].

In summary, our data indicate that FOXG1 associates to the microprocessor complex with DDX5 and that it affects mature miR200 levels. We further suggest that FOXG1 and miR200 family are both part of a multilevel network that balances the expression of a regulative subunit of PKA, PRKAR2B. Thereby, FOXG1 and also MECP2 may affect a common PKA-dependent pathway to adjust neuronal function in the hippocampus.

## Electronic Supplementary Material

Fig. S1 miR200-family and *Prkar2b* levels are not altered in adult *Foxg1*^cre/+^ cerebral cortex and olfactory bulb. (a) qRT-PCR of *Prkar2b* in adult brain cortex and olfactory bulb (OB). Results show no changes in *Prkar2b* expression in Foxg1^cre/+^ animals compared to control. (b) qRT-PCR of mature miR200b/a/429 in adult brain cortex and (c) olfactory bulb. Results show no changes in mature miR200b/a/429 levels in either regions of Foxg1^cre/+^ brains compared to control. Mean with SEM, unpaired Student’s t test. *n* = 3. (PNG 301 kb)

Fig. S2 *Prkar2b knock down* (KD) or miR200 overexpression do not alter N2a cell proliferation. Analysis of proliferation by BrdU incorporation after *Prkar2b* KD for 72 h or miR200b/a/429 overexpression (miR200 OE) for 48 h. Percentage of BrdU positive nuclei is counted as: BrdU(+)/DAPI(+)*100. Values obtained for *Prkar2b* KD or miR200 OE cells were normalized to control values (pLKO1-non-target-puro and pCX-D2eGFP-miR200 sponge, respectively). Results show no changes in BrdU incorporation. Mean with SEM, one sample t-test. n = 3. The plasmid used for *Prkar2b* KD was pLKO1-shPrkar2b-puro-GFP (CCGGTTGGAACAAACATGGATATTG CTCGAGCAATATCCATGTTTGTTCCAA TTTTTG).(PNG 5488 kb)

ESM 1 (DOCX 52 kb)

ESM 2 (XLSX 172 kb)
