# Supplementary figures and images for: FOXG1 Regulates PRKAR2B Transcriptionally and Posttranscriptionally via miR200 in the Adult Hippocampus
Source: Mol Neurobiol. 2018 Dec 11;56(7):5188–201. doi: 10.1007/s12035-018-1444-7 (PMC6647430; doi:10.1007/s12035-018-1444-7)

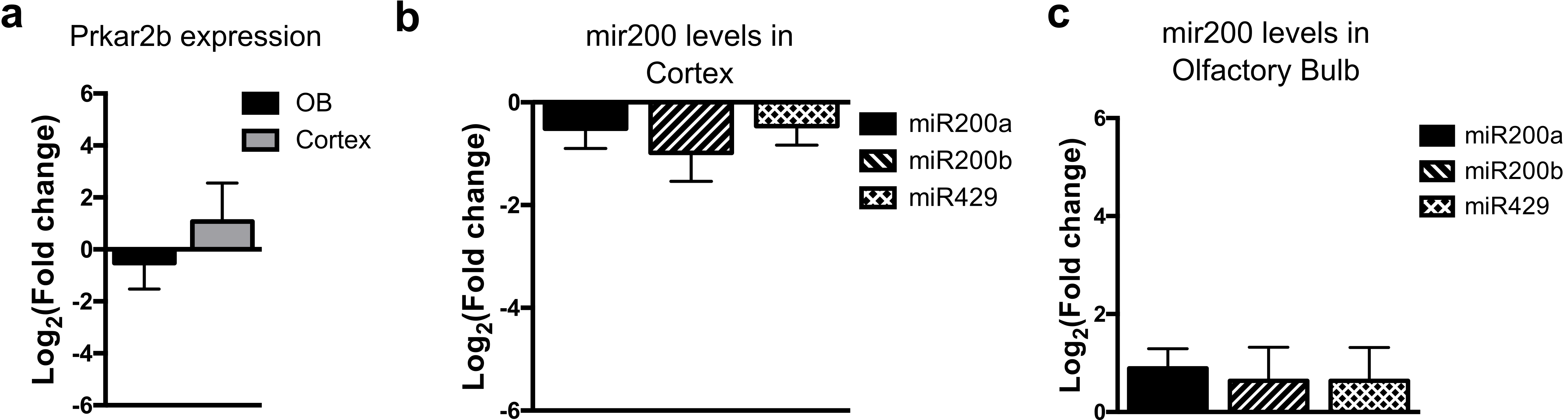

Supplement: Supplementary file 1 — miR200-family and Prkar2b levels are not altered in adult Foxg1cre/+ cerebral cortex and olfactory bulb. (a) qRT-PCR of Prkar2b in adult brain cortex and olfactory bulb (OB). Results show no changes in Prkar2b expression in Foxg1cre/+ animals compared to control. (b) qRT-PCR of mature miR200b/a/429 in adult brain cortex and (c) olfactory bulb. Results show no changes in mature miR200b/a/429 levels in either regions of Foxg1cre/+ brains compared to control. Mean with SEM, unpaired Student’s t test. n = 3. (PNG 301 kb) [file 12035_2018_1444_MOESM1_ESM.png]

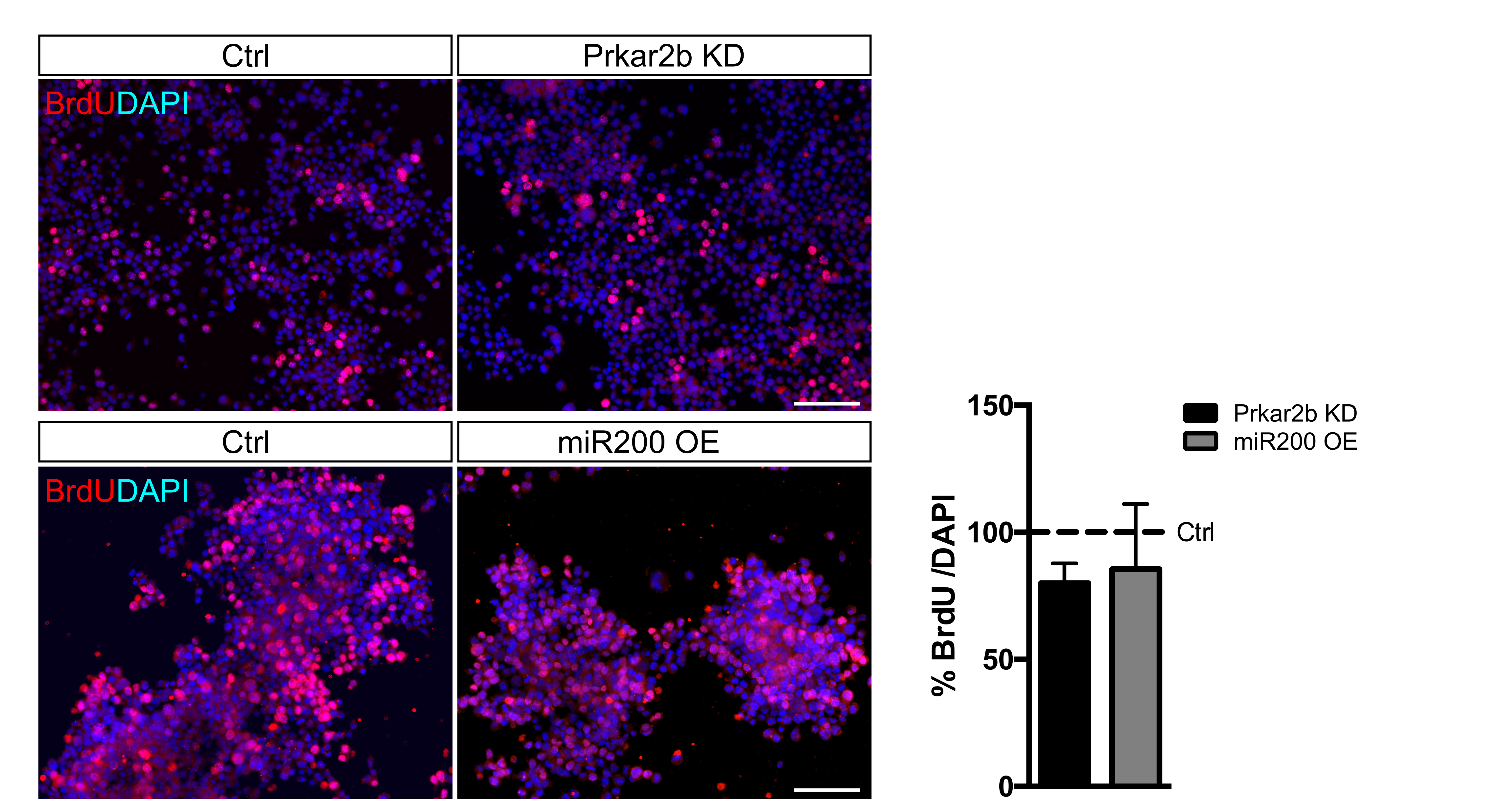

Supplement: Supplementary file 2 — Prkar2b knock down (KD) or miR200 overexpression do not alter N2a cell proliferation. Analysis of proliferation by BrdU incorporation after Prkar2b KD for 72 h or miR200b/a/429 overexpression (miR200 OE) for 48 h. Percentage of BrdU positive nuclei is counted as: BrdU(+)/DAPI(+)*100. Values obtained for Prkar2b KD or miR200 OE cells were normalized to control values (pLKO1-non-target-puro and pCX-D2eGFP-miR200 sponge, respectively). Results show no changes in BrdU incorporation. Mean with SEM, one sample t-test. n = 3. The plasmid used for Prkar2b KD was pLKO1-shPrkar2b-puro-GFP (CCGGTTGGAACAAACATGGATATTG CTCGAGCAATATCCATGTTTGTTCCAA TTTTTG).(PNG 5488 kb) [file 12035_2018_1444_MOESM2_ESM.png]
