# Supplementary material for: FOXG1 Regulates PRKAR2B Transcriptionally and Posttranscriptionally via miR200 in the Adult Hippocampus
Source: Mol Neurobiol. 2018 Dec 11;56(7):5188–201. doi: 10.1007/s12035-018-1444-7 (PMC6647430; doi:10.1007/s12035-018-1444-7)
Supplement: Supplementary file 3 — (DOCX 52 kb) [file 12035_2018_1444_MOESM3_ESM.docx]

**Supplementary Methods**

***Cell Culture and transfection***

Mouse neuroblastoma cell line, Neuro-2a (N2a) were cultured in Dulbecco’s modified Eagle’s medium (DMEM, ThermoScientific, Schwerte, Germany) supplemented with 10% fetal bovine serum (FBS, ThermoScientific), 1% non essential amino acids (NEAA, ThermoScientific), 1% L-glutamine, and 1% penicillin, streptomycin, and neomycin (PSN, ThermoScientific). Cells were maintained at 37°C, 95% relative humidity and 5% CO_2_. Cells were seeded either on coverslips, for PLA, or in 6 well plates and were transfected with Lipofectamine LTX adding a total amount of 2.5 ug plasmid according to manufactureres instructions (ThermoScientific). 10 cm dishes were transfected using calcium phosphate transfection method. For KD experiments, cells were selected with 2.4 ng/ml puromycin for 24 h.

***Plasmids***

pLenti-III-Empty-2A-GFP and pLenti-III-FOXG1-HA-2A-GFP (abmGood, Canada), pLenti-III-FOXG1-Au1-2A-GFP, pLenti-III-FOXG1-D2-Au1-2A-GFP (cloned by Dr. Gensch), pLKO-non-target-GFP, pLKO-shFoxg1-GFP and pLKO-shDdx5-GFP (Sigma). pmiRGlo-empty (Promega). pCX-miR200b/a/429/200c/141 and pCX-eGFP-miR200-sponge were generous gifted by Dr. Harold Cremer. pCX-miR200b/a/429 and pmiRGlo-miR200b/a/429 were subcloned from pCX-miR200b/a/429/200c/141 plasmid. pmiRGlo-Prkar2b-3’UTR-short*,* pmiRGlo-Prkar2b-3’UTR-inverted, pmiRGlo-Prkar2b-3’UTR-T7, pmiRGlo-5'-MCS-empty and pmirGlo-5’-MCS-Prkar2b-5’-region were cloned by GenScript USA Inc. pCMV-FOXG1-Au1 was subcloned from pLenti-III-FOXG1-Au1-2A-GFP .

***RNA isolation, reverse transcription, and quantitative real-time PCR (qRT-PCR)***

RNA was isolated from harvested cells and frozen tissue using miRNeasy kit (Qiagen) according to the manufacturer's instructions including on-column DNA digestion. 1 µg of total RNA was reverse transcribed either with RevertAid MMuLV (Fermentas, ThermoScientific) or with miScript® II RT kit (Qiagen) according to manufacturer’s protocol. mRNA samples were subjected to DNase I treatment just before cDNA synthesis with amplification grade DNase I (Sigma Aldrich) for 30 min at RT. qRT-PCR analysis for mRNA and pri-miRNA were performed on CFX-Connect Real-Time PCR detection system (Bio-Rad) using Go Taq qPCR Master Mix (Promega, Mannheim, Germany) or using Qiagen miScript SYBR® Green PCR Kit with Qiagen miScript® primer assay (miR200a/b/429 and U6) or Qiagen miScript® Precursor assay (pre-miR200a/b/429) according to manufacturer’s protocol. Primers were used at a concentration of 250 nM each. *Gapdh* or *U6* were used as reference genes. For mRNA and pri-miR200, PCR program was 3 minutes at 95°C, 40 cycles of 15 sec at 95°C and 30 sec at an annealing temperature (58°C-63°C), followed by 1 min at 95°C, 1 min at 55°C and melting curve cycle. For pre-miR200 and mature miR200, PCR program was 15 min at 95°C, 40 cycles of 15 sec at 94°C, 30 sec at 55°C and 30 sec at 70°C, followed by 1 min at 95°C, 1 min at 55°C and melting curve cycle. Primers used had an efficiency level between 85% and 110%. Primer sequences are listed in Supplementary Table S1. qRT-PCR results were analysed using the ΔΔCt method [1].

**Bioinformatics analysis of RNAseq and FOX transcription factor binding motifs**

For 6 week old *Foxg1*^cre/+^ mice hippocampal RNAseq and miRNA-200b/a/429 overexpressing N2a cells RNAseq, n = 3 and n = 2 were used respectively. Bioinformatics analysis was performed using the Freiburger Galaxy Server [2]. At first, the sequenced reads in FASTQ files were inspected using FastQC [3]. With no remarkable quality flaws from the FastQC reports, low quality bases from the 3' end were trimmed using TrimGalore [4]. For quality trimming, Phred score cut-off of 28 was used. Reads were aligned to mouse genome build mm10 using TopHat2 [5]. For Foxg1^cre/+^ samples, we set options --mate-inner-dist to 0, --mate-std-dev to 80 and --library-type to fr-firststrand; whereas default settings were used for the mir200-OE in N2a samples. For mapping both datasets --GTF option with a gene annotation model from ensemble release 79 [6] in gene transfer format were used. Later, htseq-count [7] was used to count the number of aligned reads per gene. For both datasets we set --mode to union and for *Foxg1*^cre/+^ datasets set --stranded to reverse. In the end, DESeq2 [8] was used for differential gene expression analysis. Adjusted *p* value of 0.05 or less as the significant threshold was chosen for differentially expressed genes.

Small RNA-Seq was performed on an Illumina HiSeq 2000 system. Small RNA libraries were prepared from 1 µg total RNA using the Illumina TruSeq Small RNA Sample Preparation kit. For processing of sequencing data a customized in-house software pipeline was used. Quality check and demultiplexing were performed using the CASAVA 1.8.2 software (Illumina). We trimmed the 3' adapters and filtered out the reads with the minimum length of 15 nucleotides using cutadapt. We first map the reads to the reference genome created from microRNA sequences. Remaining unmapped reads were then mapped to mouse genome. We used rna-STAR for all the mapping. We allowed no mismatches for the reads <25b, one mismatch for reads between 26b to 33b. We mapped all the reads in the non-splice-junction-aware mode. For comparison of miRNA expression between samples, a differential expression analysis was performed using R, DESeq2 and RUVseq package. miRNAs were considered to be differentially expressed with an adjusted p-value below 0.05.

To identify putative binding sites of forkhead box binding sites, we downloaded all mouse FOX transcription factor binding profiles from JASPAR database [9]. We subsequently used the tool FIMO [10] with default settings to search for the FOX motifs on 1000 bp upstream of the mir200b/a/429 gene cluster.

GO term and KEGG pathway analyses were performed with DAVID [11, 12]. The 34 overlapping genes from miR200-overexpression and *Foxg1^cre/+^* RNA-Seq were used for GO term analyses for biological processes and cellular compartments using the ''official gene names’’ and "mus musculus" as species. To identify miR200 targets among the 34 targets, the following miRNA target prediction tools were used: Targetscan v6.2 [13], miRanda [14], miRDB [15], MicroCosm v5 [16] .

***Luciferase Assay***

N2a cells were transfected with pmiRGlo-miR200b/a/429 for DROSHA activity assay or with pmiRGlo-Prkar2b-3’UTR-short*,* pmiRGlo-Prkar2b-3’UTR-Invert or pmiRGlo-Prkar2b-3’UTR-T_7_ for miR200 target validation experiment. For FOXG1 activity on *Prkar2b* promoter we used the plasmids pmirGlo-5’-MCS-empty
 and pmirGlo-5’-MCS-Prkar2b-5’-region. Cells were harvested 48 h after transfection with 1X Passive Lysis Buffer (Promega). The luciferase assay was performed with the Dual Luciferase System Kit (Promega) according to manufacturer’s instructions. Shortly, 5 µl cell lysate was first incubated with LARII as substrate for firefly luciferase followed by Stop&Glo to inhibit firefly luciferase activity and as a substrate for the renilla luciferase. Luminescence intensity was measured with a 2 s delay for 10 s, with the Glomax96 luminometer. Firefly luciferase activity was normalized to renilla luciferase activity to calculate relative luciferase activity of each condition.

***SILAC and mass spectrometry***

For HA-co-IP, one 6-well plate with 250000 cells per well were transfected either with pLenti3-Foxg1-HA-T2A-eGFP (abmGood, Canada) or pLenti3-Foxg1-Au1-T2A-eGFP. Cells were lysed in co-IP buffer (100 mM NaCl, 20 mM Tris, 1 mM EDTA, 0.5% NP40-alternative, pH7.4) supplemented with protease inhibitor (cOmplete Protease Inhibitor Cocktail, Roche-Diagnostics, Mannheim, Germany). Protein amounts of both conditions were estimated by Bradford reagent (BioRad, Munich, Germany). 1.4 mg of each condition was precleared for 1~h with sepharose beads (Protein A Sepharose Cl-4B, GE Healthcare), before HA-co-IP was performed with 70 µl of HA-coupled sepharose beads (#3956, Cell-Signaling, Frankfurt a. M., Germany) over night. Antigen-coupled beads were washed 3 times in co-IP buffer. After the last washing, HA-IP and MOCK-IP were pooled and resuspended in 60 µl 1x Laemmli buffer.

Samples for mass spectroscopy were prepared with 1 mM DTT for 5 min at 95⁰C and alkylated using 5.5 mM iodacetamide for 30 min at 25⁰C. Protein mixtures were separated by SDS-PAGE (4-12% Bis-Tris mini gradient gel) and gel lanes were cut into 10 equal slices. Gel fractions were in-gel digested using trypsin (Promega, Mannheim, Germany) [17]. Digests were performed overnight at 37°C in 0.05 M NH4HCO3 (pH 8). About 0.1 µg of protease was used for each gel band. Peptides were extracted from the gel slices with ethanol and resulting peptide mixtures were processed on STAGE tips as described [18].

Samples analyzed by MS were measured on LTQ Orbitrap XL mass spectrometer (ThermoFisherScientific, Bremen, Germany) coupled to an Agilent 1200 nanoflow-HPLC (Agilent Technologies GmbH, Waldbronn, Germany). HPLC-column tips (fused silica) with 75 µm inner diameter were self-packed with Reprosil-Pur 120 ODS-3 to a length of 20 cm. No pre-column was used. Peptides were injected at a flow of 500 nl/min in 92% buffer A (0.5% acetic acid in HPLC gradient grade water) and 2% buffer B (0.5% acetic acid in 80% acetonitrile, 20% water). Separation was achieved by a linear gradient from 10% to 30% of buffer B at a flow rate of 250 nl/min. The mass spectrometer was operated in the data-dependent mode and switched automatically between MS (max. of 1 x10 ions) and MS/MS. Each MS scan was followed by a maximum of five MS/MS scans in the linear ion trap using normalized collision energy of 35% and a target value of 5,000. Parent ions with a charge states of z = 1 and unassigned charge states were excluded from fragmentation. The mass range for MS was m/z = 370 to 2,000. The resolution was set to 60,000. MS parameters were as follows: spray voltage 2.3 kV; no sheath and auxiliary gas flow; ion transfer tube temperature 125°C. Software Xcalibur (Thermo Scientific) and Mascot Daemon version 2.4.0 (Matrix Science, London, UK) were used for data acquisition and processing.

The MS raw data files were uploaded into the MaxQuant software version 1.4.1.2 [19], which performs peak and SILAC-pair detection, generates peak lists of mass error corrected peptides and data base searches. A full-length mouse database containing common contaminants, such as keratins and enzymes used for in-gel digestion, was employed, carbamidomethylcysteine was set as fixed modification and methionine oxidation and protein amino-terminal acetylation were set as variable modifications. Double SILAC was chosen as quantification mode. Three miss cleavages were allowed, enzyme specificity was trypsin/P+DP, and the MS/MS tolerance was set to 0.5 Da. The average mass precision of identified peptides was in general less than 1 ppm after recalibration. Peptide lists were further used by MaxQuant to identify and relatively quantify proteins using the following parameters: peptide, and protein false discovery rates (FDR) were set to 0.01, maximum peptide posterior error probability (PEP) was set to 0.1, minimum peptide length was set to 6, minimum number peptides for identification and quantitation of proteins was set to two, of which one must be unique, and identified proteins have been re-quantified. The “match-between-run” option (2 min) was used.

***Immunoprecipitation***

Tissue or N2a cells were lysed in co-IP buffer (100 mM NaCl, 20 mM Tris, 1 mM EDTA, 0.5% NP40-alternative, pH 7.4) supplemented with protease inhibitor (Roche) and lysed by incubation for 30 min on ice, triturating every 10 min 20 times. After centrifugation (10 min, 13000 rpm) the supernatant was collected. Protein concentrations were determined with Bradford reagent (Bio-Rad). 5% input was saved and equal amounts of protein were used for MOCK and all co-IPs. Protein G Dynabeads (10004D, ThermoScientific) were coupled for 1 h at room temperature and 1 h at 4°C with Co-IP antibodies or control IgG antibody (rabbit IgG kch-504-250, Diagenode, Seraing, Belgium). Cell lysates were blocked with Protein G Dynabeads for 1 h at 4°C, subsequently transferred to antibody-coupled bead and incubated while rotating over night at 4°C. Antigen-coupled beads were washed 3 times with co-IP buffer before they were resuspended in 30 µl 1x laemmli buffer. 5% input and the complete Co-IP sample were used for immunoblotting.

***Immunoblotting***

Protein samples for WB were prepared as described for the co-IP samples. Protein or co-IP samples were loaded either on 8% or 10% SDS-polyacrylamide gels and run at 120V for 1.5 h. Proteins were transferred to PVDF membranes (Trans-blot Turbo Transfer Pack) using the Trans-blot Turbo Transfer System (Bio-Rad) following the manufacturer’s instructions. Membranes were blocked with 5% BSA in TBS-T (blocking buffer) for 1 h and incubated overnight with primary antibodies (diluted in blocking buffer). Membranes were washed, incubated with secondary antibodies for 1 h and detected using Femto substrates (Thermo Scientific) and LAS ImageQuant System (GE Healthcare, Little Chalfont, UK).

***Cell fractionation***

For protein and co-IP, cytoplasm, nucleoplasm and chromatin were fractionated according to the protocol reported in [20].

***Proximity ligation assay (PLA)***

N2a cells were fixed in 4% PFA. Cells were permeabilized for 15 min with 0.1% Triton-X100, before incubation with the Duolink blocking solution for 1 h. Cells were incubated with primary antibodies diluted in Duolink antibody diluent solution over night at 4°C. After washing the cells, they were first incubated with PLA-RED Probes for 1h at 37°C, then with ligation solution for 30 min at 37°C and finally amplification solution was added for 100 min at 37°C. For the following immunocytochemistry, cells were blocked again with Duolink blocking solution/0.1% Triton-X100 for 1 h before they were incubated with the primary Lamin B1 antibody in the blocking solution over night at 4°C. Following washing with PBS, cells were incubated with the donkey-anti-rabbit-488 (1:500, 711-545-152, Dianova) for 1 h at room temperature. Before mounting coverslips with fluorescent mounting medium (#S3023, DAKO, Jena, Germany), nuclei were stained with DAPI.

***RNA immunoprecipitation (RIP)***

N2a cells were cultured in 10 cm dishes (one 10 cm dish was used per RIP) and after 48 h of transfection, cells were collected and lysed using 750 µl RIPA buffer (150 mM NaCl, 1% NP-40, 0.5% Sodium deoxycholate, 0.1% SDS, 50 mM Tris-HCl (pH 7.4), 1 mM EDTA). 250 µl lysate were used for FOXG1-Au1 or DDX5 RIP and 250 µl lysate for IgG RIP. The remaining lysate was saved as input. 7 µl of anti-Au1, anti-DDX5 or an appropriate IgG antibody was incubated with Protein G Dynabeads in RIPA for 2 hr at RT. The cell lysates were incubated with the antibody coupled beads overnight at 4°C. After incubation, the beads were washed four times in high salt RIPA buffer (1 M NaCl, 1% NP-40, 0.5% Sodium deoxycholate, 0.1% SDS, 50 mM Tris-HCl (pH 7.4), 1 mM EDTA), followed by a final wash in 1 ml PBS. 100 µl of beads were collected for protein analysis by immunoblot and 900 µl of beads were used for RNA extraction using Qiagen miRNeasy kit according to the manufacturer’s protocol.

## *miRNA analysis by Northern hybridization*

The separation of RNA samples enriched for small RNAs via denaturing polyacrylamide gel electrophoresis and their analysis by Northern hybridization was performed as described [21] with the following variations. 4-5 µg of small RNA per lane were separated on polyacrylamide (PAA)-urea minigels (15% PAA, 0.5 g/ml urea, 1x Tris-Borate-EDTA (TBE) buffer), electroblotted and cross-linked onto positivated Porablot NY plus nylon membrane (Macherey-Nagel GmbH & Co. KG). The RNA sizes were estimated using the microRNA Marker (NEB). For the hybridization of the U6 snRNA, membranes were prehybridized for 60 min at 62°C, for the detection of miRNAs and the marker at 45°C with hybridization buffer (50% deionized formamide, 7% SDS, 250 mM sodium chloride, 120 mM sodium phosphate, pH 7.2) under continuous rotation. Probes (for the detection of miR429, mir200a, miR200b and U6 snRNA) were generated by *in vitro* transcription using *mir*Vana^TM^ miRNA probe construction kit (Thermo Fischer Scientific), while the microRNA marker was detected by hybridization with probe (5´-AAATCTCAACCAGCCACTGCT-3´-Biotin) supplied by NEB. The probes against microRNA marker were 5’-end-labeled using 50 µCi [γ32P] ATP (3.000 Ci/mmol, Hartmann Analytic) and 20 U of T4 polynucleotide kinase (Thermo Fisher Scientific) for 30 min at 37°C. Membranes were hybridized at 62°C (U6 snRNA) or 45°C (all other probes) over night and washed at 57°C (U6 snRNA) or 40°C (other probes) with washing solutions I (2x SSC and 1% SDS), II (1x SSC and 0.5% SDS) and III (0.1x SSC and 0.1% SDS) for 10 min each. The signals were detected with a storage phosphor screen (Kodak) and a **GE Typhoon FLA 9500 imaging system**.

***Mouse hippocampus dissection, culture of neurons and viral transduction***

NMRI (Charles River) hippocampi of P0 embryos were dissected and collected in 5 ml Hanks’ Balanced Salt (HBSS, Fisher Scientific, Schwerte, Germany) and dissociated in 0.25% Trypsin/EDTA (Fisher Scientific) at 37 °C for 10 min. Dissociation was stopped by adding NB-complete medium and 10% fetal bovine serum (FBS, Fisher Scientific). Cells were collected by centrifugation and cultured in NB-complete medium (Neurobasal medium (Fisher Scientific) supplemented with B27 (Fisher Scientific), L-glutamine (0.5 mM, Fisher Scientific), penicillin-streptomycin-neomycin (PSN, Fisher Scientific), apo-transferrin (5 µg/ml, Sigma, München, Germany), superoxid-dismutase (0.8 µg/ml, Sigma) and glutathione (1 µg/ml, Sigma)). Cells were always seeded on poly-ornithine (0.1 mg/ml, Sigma) and laminin (1 µg/ml, Sigma) wells of 24 well plates.

Lentiviral particles using pLKO1-shDdx5-puro, pLKO1-shFoxg1-puro-GFP or pLKO1-non-target-puro (Sigma) were prepared according to the protocol described previously [22, 23]. On day *in vitro* (DIV) 2 cells were transfected with lentiviral particles. At DIV5, transduced cells were selected with 0.3 µg/ml puromycin and cell proliferation was inhibited by addition of 2 µM AraC, while performing a half medium change. Medium was changed again at DIV9 including 2 µM AraC. Cells were harvested at DIV11 in Qiazol reagent and used for RNA extraction.

***BrdU (Bromodeoxyuridine) incorporation and immunofluorescence***

For proliferation assays, N2A cells were transfected with the plasmids indicated in the figures and a 1 h BrdU pulse (Roche BrdU Kit) was given before fixation. Cells were fixed with 4% PFA for 20 min at room temperature. For the BrdU antigen retrieval, fixed cells were treated with 1N HCl during 30 min followed by two washes with Boratbuffer (150 mM H3BO3, pH 8,4) of 10 min each for neutralization. Cells were then permeabilised and blocked in 10% horse serum / 0.1% Triton-X100/PBS for 1 hour and incubation with anti-BrdU antibody (1:200, sheep, ab1893, abcam) was perfomed over night at 4°C in blocking solution. Cells were washed 3 times in PBS and then incubated with fluorophore-coupled secondary antibodies in blocking solution at room temperature. After 3 washes with PBS, cells were incubated for 1 min in DAPI solution and washed 3 more times in PBS. Coverslips were mounted on glass slides with fluorescent mounting medium (#S3023, DAKO, Jena, Germany). Images were obtained using an Axioplan M2 fluorescent microscope (Zeiss) and processed with FIJI (ImageJ, v. 2.0.0-rc-43/1.51d)[24][23][22][21][20][18][17] and , Inkscape (v. 0.91).

**References**

1. Livak KJ, Schmittgen TD (2001) Analysis of Relative Gene Expression Data Using Real-Time Quantitative PCR and the 2−ΔΔCT Method. Methods 25:402–408. https://doi.org/10.1006/meth.2001.1262

2. Grüning BA, Fallmann J, Yusuf D, et al (2017) The RNA workbench: best practices for RNA and high-throughput sequencing bioinformatics in Galaxy. Nucleic Acids Res 45:W560–W566. https://doi.org/10.1093/nar/gkx409

3. Andrews S FastQC A Quality Control tool for High Throughput Sequence Data

4. Krueger F A wrapper tool around Cutadapt and FastQC to consistently apply quality and adapter trimming to FastQ files, with some extra functionality for MspI-digested RRBS-type (Reduced Representation Bisufite-Seq) libraries.

5. Kim D, Pertea G, Trapnell C, et al (2013) TopHat2: accurate alignment of transcriptomes in the presence of insertions, deletions and gene fusions. Genome Biol 14:R36. https://doi.org/10.1186/gb-2013-14-4-r36

6. Yates A, Akanni W, Amode MR, et al (2015) Ensembl 2016. Nucleic Acids Res 44:D710. https://doi.org/10.1093/nar/gkv1157

7. Anders S, Pyl PT, Huber W (2014) HTSeq—a Python framework to work with high-throughput sequencing data. Bioinformatics 31:166. https://doi.org/10.1093/bioinformatics/btu638

8. Love MI, Huber W, Anders S (2014) Moderated estimation of fold change and dispersion for RNA-seq data with DESeq2. Genome Biol 15:550. https://doi.org/10.1186/s13059-014-0550-8

9. Mathelier A, Fornes O, Arenillas DJ, et al (2016) JASPAR 2016: a major expansion and update of the open-access database of transcription factor binding profiles. Nucleic Acids Res 44:D110–D115. https://doi.org/10.1093/nar/gkv1176

10. Grant CE, Bailey TL, Noble WS (2011) FIMO: scanning for occurrences of a given motif. Bioinformatics 27:1017–1018. https://doi.org/10.1093/bioinformatics/btr064

11. Huang DW, Sherman BT, Lempicki RA (2009) Bioinformatics enrichment tools: paths toward the comprehensive functional analysis of large gene lists. Nucleic Acids Res 37:1–13. https://doi.org/10.1093/nar/gkn923

12. Huang DW, Sherman BT, Lempicki RA (2009) Systematic and integrative analysis of large gene lists using DAVID bioinformatics resources. Nat Protoc 4:44–57. https://doi.org/10.1038/nprot.2008.211

13. Lewis BP, Shih I -hun., Jones-Rhoades MW, et al (2003) Prediction of Mammalian MicroRNA Targets. Cell 115:787–798. https://doi.org/10.1016/S0092-8674(03)01018-3

14. Betel D, Wilson M, Gabow A, et al (2008) The microRNA.org resource: targets and expression. Nucleic Acids Res 36:D149–D153. https://doi.org/10.1093/nar/gkm995

15. Wong N, Wang X (2015) miRDB: an online resource for microRNA target prediction and functional annotations. Nucleic Acids Res 43:D146–D152. https://doi.org/10.1093/nar/gku1104

16. Griffiths-Jones S, Saini HK, van Dongen S, Enright AJ (2008) miRBase: tools for microRNA genomics. Nucleic Acids Res 36:D154–D158. https://doi.org/10.1093/nar/gkm952

17. Shevchenko A, Tomas H, Havli J, et al (2007) In-gel digestion for mass spectrometric characterization of proteins and proteomes. Nat Protoc 1:2856–2860. https://doi.org/10.1038/nprot.2006.468

18. Rappsilber J, Mann M, Ishihama Y (2007) Protocol for micro-purification, enrichment, pre-fractionation and storage of peptides for proteomics using StageTips. Nat Protoc 2:1896–1906. https://doi.org/10.1038/nprot.2007.261

19. Cox J, Mann M (2008) MaxQuant enables high peptide identification rates, individualized p.p.b.-range mass accuracies and proteome-wide protein quantification. Nat Biotechnol 26:1367–1372. https://doi.org/10.1038/nbt.1511

20. Vance KW, Sansom SN, Lee S, et al (2014) The long non‐coding RNA Paupar regulates the expression of both local and distal genes. EMBO J e201386225. https://doi.org/10.1002/embj.201386225

21. Behler J, Sharma K, Wilde A, et al (2018) The host-encoded RNase E endonuclease as the maturation enzyme of a CRISPR-Cas subtype III-B system. Nature Microbiol in press

22. Hellbach N, Weise SC, Vezzali R, et al (2014) Neural deletion of Tgfbr2 impairs angiogenesis through an altered secretome. Hum Mol Genet 23:6177–6190. https://doi.org/10.1093/hmg/ddu338

23. Vezzali R, Weise SC, Hellbach N, et al (2016) The FOXG1/FOXO/SMAD network balances proliferation and differentiation of cortical progenitors and activates Kcnh3 expression in mature neurons. Oncotarget 5:

24. Schindelin J, Arganda-Carreras I, Frise E, et al (2012) Fiji: an open-source platform for biological-image analysis. Nat Methods 9:676–682. https://doi.org/10.1038/nmeth.2019

**Supplementary Table S1: qRT-PCR Primers**

| Ddx5_forq | TCCAGAGGGCTAGATGTGGAA |
| --- | --- |
| Ddx5_revq | TGCCTGTTTTGGTACTGCGA |
| Enc1_forq | TACTGCATTCGTCAGCACCT |
| Enc1_revq | ATGACATCTCATCTCGCCGT |
| Foxg1_forq | AATGACTTCGCAGACCAGCA |
| Foxg1_revq | CCGGACAGTCCTGTCGTAAA |
| Gapdh_forq | CGGCCGCATCTTCTTGTG |
| Gapdh_revq | TGACCAGGCGCCCAATAC |
| Homer3_forq | CAGTCGAGCCAACACTGTCT |
| Homer3_revq | AGATTTCTCTCGAGCCAGCC |
| Itpka_forq | ATCTGCTGAGCGACAGTGAG |
| Itpka_revq | GCAGATTGACCATGGTACGG |
| Nrp2_forq | GACGATCGGGAGAGATTTCCA |
| Nrp2_revq | AATCCTCACCTGCAAAAGCTG |
| Olfml2b_forq | GCTGACAGCCGAGATTTGAAG |
| Olfml2b_revq | GTGGTAACCGAATGCAGCTT |
| pri-miR200a_forq | CGGACAGTGCTGGATTTCTT |
| pri-miR200a_revq | CAGGAGGACAAGTGTGTATCATC |
| pri-miR200b_forq | TGATCTCTAATACTGCCTGGTAATG |
| pri-miR200b_revq | CCATAGCCCTACCTTGGATAAG |
| pri-miR429_forq | CCTTCCCTCTACAGGTATCAAATC |
| pri-miR429_revq | GACGGCATTACCAGACAGTATTA |
| Prkar2b_forq | GCAAGAGGCTTGCAAAGACA |
| Prkar2b_revq | CGTGTTCCCCTTCTTTGACC |
| Serpinf1_forq | ACGGCTTGGACTCTGATCTC |
| Serpinf1_revq | TCAAGTTCTGGGTCACGGTC |
| Sqle_forq | TCACCATGGCCGATTCATCA |
| Sqle_revq | CCTTGTATTGCACGCCGATT |
| Stc2_forq | TTTCTGCACAACGCTGGAAA |
| Stc2_revq | CCAAATTTATGACGCAGGGCA |
| Tmem108_forq | GCATGCAGAAGTAAGCGTGT |
| Tmem108_revq | CCTGCTGGTCCTCCAGTTTA |
| Ybx3_forq | TACAGACGCGGCTACTATGG |
| Ybx3_revq | GGGCTCAAATCCTTCACTGC |
| Zdhhc15_forq | GAAGCAGATGCTTGTGGACAT |
| Zdhhc15_revq | TTGATTAGATGGCACCGGTCA |
| Zeb1_forq | CTGCTCCCTGTGCAGTTACA |
| Zeb1_revq | CTTGAACTTGCGGTTTCCCC |
| Zfp462_forq | GGCCACGGATTGTCAGTCTCC |
| Zfp462_revq | GGGGCTCCGAGTAAGAGGAGT |
